# IFI16 phase separation via multi-phosphorylation drives innate immune signaling

**DOI:** 10.1093/nar/gkad449

**Published:** 2023-06-07

**Authors:** Dawei Liu, Krystal K Lum, Nicholas Treen, Corazón T Núñez, Jinhang Yang, Timothy R Howard, Michael Levine, Ileana M Cristea

**Affiliations:** Department of Molecular Biology, Princeton University, Princeton, NJ 08544, USA; Department of Molecular Biology, Princeton University, Princeton, NJ 08544, USA; Lewis-Sigler Institute for Integrative Genomics, Princeton University, Princeton, NJ 08544, USA; Department of Molecular Biology, Princeton University, Princeton, NJ 08544, USA; Department of Molecular Biology, Princeton University, Princeton, NJ 08544, USA; Department of Molecular Biology, Princeton University, Princeton, NJ 08544, USA; Department of Molecular Biology, Princeton University, Princeton, NJ 08544, USA; Lewis-Sigler Institute for Integrative Genomics, Princeton University, Princeton, NJ 08544, USA; Department of Molecular Biology, Princeton University, Princeton, NJ 08544, USA

## Abstract

The interferon inducible protein 16 (IFI16) is a prominent sensor of nuclear pathogenic DNA, initiating innate immune signaling and suppressing viral transcription. However, little is known about mechanisms that initiate IFI16 antiviral functions or its regulation within the host DNA-filled nucleus. Here, we provide *in vitro* and *in vivo* evidence to establish that IFI16 undergoes liquid–liquid phase separation (LLPS) nucleated by DNA. IFI16 binding to viral DNA initiates LLPS and induction of cytokines during herpes simplex virus type 1 (HSV-1) infection. Multiple phosphorylation sites within an intrinsically disordered region (IDR) function combinatorially to activate IFI16 LLPS, facilitating filamentation. Regulated by CDK2 and GSK3β, IDR phosphorylation provides a toggle between active and inactive IFI16 and the decoupling of IFI16-mediated cytokine expression from repression of viral transcription. These findings show how IFI16 switch-like phase transitions are achieved with temporal resolution for immune signaling and, more broadly, the multi-layered regulation of nuclear DNA sensors.

## Graphical Abstract

**Graphical Abstract:**
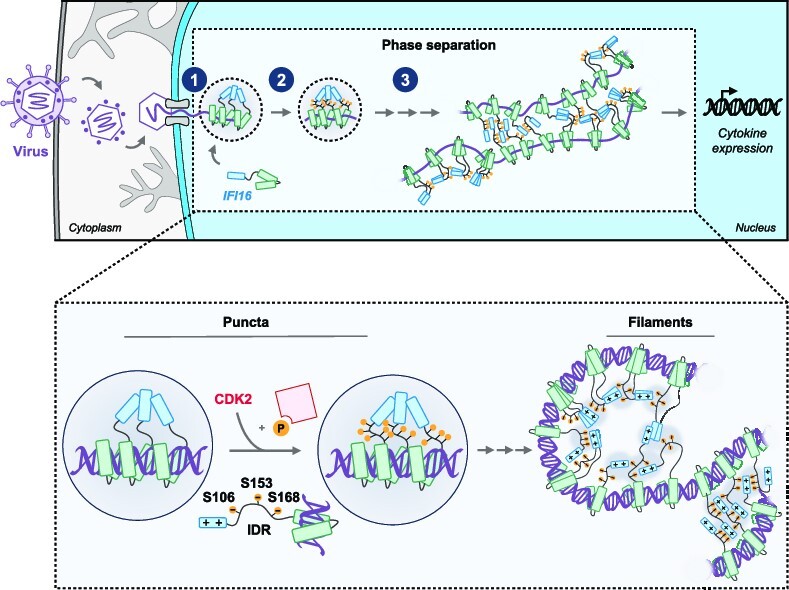

## INTRODUCTION

Throughout evolution, mammalian cells have developed a complex intrinsic and innate immune system for defense against pathogens by using pattern recognition receptors (PRRs) to detect pathogen associated molecular patterns (PAMPs). During viral infection, the nucleic acid that comprises the viral genome is a prototypic PAMP that can be recognized by host PRR sensors of viral DNA or RNA (1–3). Upon detection and direct binding, these sensors initiate rapid and extensive signaling cascades that can promote the expression of type I interferons (IFN), pro-inflammatory cytokines, and interferon-stimulated genes. The functions of these signaling molecules during an infection span a broad range of immune responses, which culminate in the intra- and inter-cellular restriction of virus replication and spread. To exert such functions, a paradigm has emerged supporting the universal formation of higher-order PRR assemblies and supramolecular complexes of nanometer to micrometer scales for innate immune signal amplification (4).

A class of viral nucleic acid sensors suggested to form multimeric platforms for amplifying immune signals, which we and others have characterized over the past decade, are those residing in the nucleus (5–9). A prominent nuclear sensor that arose from these studies is the adaptively evolved interferon-inducible protein 16 (IFI16) (6,8,10,11). IFI16 has been shown to bind to the DNA genomes of herpesviruses, including herpes simplex virus type 1 (HSV-1), human cytomegalovirus (HCMV) (11), Kaposi's sarcoma-associated herpesvirus (KSHV) (12), as well as to DNA intermediates of retrovirus human immunodeficiency virus 1 (HIV-1) (13). Upon binding to viral DNA via its two HIN200 domains, IFI16 undergoes cooperative oligomerization mediated by the N-terminal PYRIN domain (PY) and induces two types of antiviral responses—the induction of antiviral cytokines and suppression of viral gene expression (14–20).

The prevailing hypothesis indicates that IFI16-mediated cytokine induction occurs primarily through signaling to a central cytoplasmic axis, where the endoplasmic reticulum (ER)-resident protein stimulator of interferon genes (STING) activates TANK-binding kinase 1 (TBK-1). TBK-1 subsequently phosphorylates the interferon regulatory factor 3 (IRF3), promoting its dimerization and translocation into the nucleus, where it serves as a transcription factor to induce expression of IFN-stimulated genes and cytokines (21–23). Despite this knowledge, how IFI16 structures are dynamically reorganized for immune signal nucleation and amplification is less clear and has remained as an acute broader question for PRR sensing and autoimmunity.

We have previously shown that PY-mediated oligomerization is critical for the ability of IFI16 to induce antiviral cytokines and reduce viral spread (24). The importance of IFI16 oligomerization in promoting its antiviral functions is also substantiated by the existence of multiple virus immune evasion mechanisms that inactivate this property. The HCMV viral tegument protein pUL83 binds IFI16 PY and inhibit its oligomerization (25), whereas HSV-1 encodes a viral E3 ubiquitin ligase ICP0 that targets IFI16 PY and promotes IFI16 degradation (8,14). A key characteristic of IFI16 oligomerization is its temporally distinct structural states during infection. During early stages of infection, our group and others have observed that IFI16 rapidly moves to the incoming viral DNA at the nuclear periphery, followed by a highly dynamic on/off process of puncta formation (14,26,27). The second stage is manifested as puncta localizing to nucleoplasmic regions containing nuclear bodies and viral replication compartments (14,17,26,28). This nuclear localization is followed by the eventual degradation of IFI16 in the context of wild-type HSV-1 infection (8,28–30). In contrast, when cells are infected with ICP0-null HSV-1 virus and exhibit heightened antiviral cytokine levels, IFI16 oligomerization has been shown to expand into filamentous structures that can recruit host restriction factors to suppress viral replication (20,29,31). It was also established that these filaments assembled cooperatively, and the strength of cooperativity is positively correlated with the length of DNA (16).

Despite such advances, many molecular aspects of IFI16 oligomerization remain unclear. Little is known about the biophysical properties of IFI16 oligomers that govern their morphological changes upon DNA sensing. It is also unclear how IFI16 oligomers are regulated to prevent autoimmune activation due to the presence of host DNA. Given that IFI16 cooperative binding *in vitro* was shown to be dependent on DNA length, it has been speculated that the presence of nucleosomes could prevent IFI16 diffusion along host DNA and oligomerization (17), thereby distinguishing self from non-self DNA (16,31). In addition, it remains unknown whether the distinct phases of IFI16, from the dynamic puncta at the nuclear periphery to the stable filaments, have distinct biophysical properties and serve different functions. The mechanisms underlying the transition of IFI16 through these stages of host antiviral response remain to be elucidated.

Here, we provide *in vitro* and *in vivo* evidence that the formation of IFI16 puncta and filaments is mediated by liquid–liquid phase separation (LLPS), which is nucleated by interaction with double stranded DNA (dsDNA). Using mutagenesis and microscopy, we have identified an intrinsically disordered region (IDR) on IFI16 that mediates its LLPS. We show that multiple phosphorylation sites within the IDR function in a combinatorial manner to promote IFI16 transition through phase separation stages. Results from mutagenesis and truncation of IFI16 domains lead us to propose a model in which IFI16 filaments are assembled through the end-to-end binding of IFI16 PY and IDR, mediated by PY charged residues and IDR phosphorylations. We further establish that IFI16 LLPS is critical for its induction of antiviral cytokines and inhibition of viral spread, but not for the initial suppression of virus gene expression, providing an example of functional decoupling of the two antiviral functions of IFI16. Additionally, using biochemical enrichment, bioinformatics, and mass spectrometry, we identified CDK2 and GSK3β as kinases present at the nuclear periphery during infection and contributing to the IFI16 IDR phosphorylation. These observations provide a unifying explanation for how IFI16 switch-like phase separations, from puncta on/off kinetics to filamentation, are achieved with high temporal resolution for immune signaling initiation and maintenance. IFI16 oligomerization as dictated by its phosphorylation status serves as a biophysical rheostat that toggles phases of innate immune activity. Overall, our findings uncover biophysical properties of mammalian PRR sensors in the nucleus, providing key insights into the tight regulation of IFI16 and innate immunity.

## MATERIALS AND METHODS

See Tables 1, 2 and 3.

**Table 1.** Reagents

REAGENT or RESOURCE	SOURCE	IDENTIFIER
**Antibodies**		
Anti-ICP4, Mouse	Santa Cruz Biotechnology	sc-69809
Anti-alpha-tubulin, Mouse	Sigma Aldrich	T6199
Anti-GAPDH, Rabbit	Cell Signaling	D16H11
Anti-IFI16, Mouse	Santa Cruz Biotechnology	sc-8023
Anti-IFI16, Mouse	Sigma Aldrich	WH0003428M3
Anti-GFP, Mouse	Sigma Aldrich	11814460001
Anti-NUP153, Mouse	Abcam	ab24700
Anti-histone H3, Rabbit	Abcam	ab1791
Anti-calreticulin, Rabbit	Abcam	ab2907
GFP-Trap Magnetic Agarose	Chromotek	gtma-20
Alexa Fluor secondary antibodies	Thermo Fisher Scientific	A-11001, A-11004, A21141, A21124
**Chemicals, inhibitors, and recombinant proteins**		
X-tremeGENE HP DNA transfection reagent	Sigma Aldrich	6366236001
Prolong Diamond Antifade Mountant	Thermo Fisher Scientific	P36961
Immobilon-FL PVDF membranes	Millipore Sigma	IPFL00010
TCEP	ThermoFisher Scientific	77720
S-TRAP Micro Spin Columns	Protifi	CO2-micro-10
DAPI	Thermo Fisher Scientific	62248
AT7519	Cayman Chemical	16231
1,6-Hexanediol	Sigma Aldrich	240117
Recombinant cGAS	Cayman Chemical	22810
Recombinant IFI16	OriGene	TP302193
TrueCut Cas9 Protein v2	Invitrogen	A36498
Recombinant Cdk2/Cyclin E	Sigma Aldrich	14–475
Recombinant DNA-PK	Life Technologies Corporation	PV5866
Recombinant GSK3 beta	Abcam	ab60863
**Critical commercial assays, kits and reagents**		
BCA Assay	Thermo Fisher Scientific	23225
Roche *In Situ* Cell Death Detection Kit – Fluorescein TUNEL Assay	Sigma Aldrich	11684795910
Alexa Fluor™ 488 Protein Labeling Kit	Thermo Fisher Scientific	A10235
HiScribe® T7 High Yield RNA Synthesis Kit	New England BioLabs	E2040S
Applied Biosystems PowerUp SYBR Green Master Mix	Thermo Fisher Scientific	A25778
**Oligonucleotides**		
Cy5-VACV70mer F	IDT	5′- CCATCAGAAAGAGGTTTAATATTTTTGTGAGACCATCGAAGAGAGAAAGAGATAAAACTTTTTTACGACT
Cy5-VACV70mer R	IDT	5′- AGTCGTAAAAAAGTTTTATCTCTTTCTCTCTTCGATGGTCTCACAAAAATATTAAACCTCTTTCTGATGG
*isg54* qPCR primer F	IDT	5′-ACGGTATGCTTGGAACGATTG
*isg54* qPCR primer R	IDT	5′- AACCCAGAGTGTGGCTGATG
*isg56* qPCR primer F	IDT	5′-AAGGCAGGCTGTCCGCTTA
*isg56* qPCR primer R	IDT	5′-TCCTGTCCTTCATCCTGAAGCT
*icp4* qPCR primer F	IDT	5′-GCGTCGTCGAGGTCGT
*icp8* qPCR primer R	IDT	5′-CGCGGAGACGGAGGAG
*icp4* qPCR primer F	IDT	5′-GTGGTTACCGAGGGCTTCAA
*icp8* qPCR primer R	IDT	5′-GTTACCTTGTCCGAGCCTCC
*ul5* qPCR primer F	IDT	5′-TCGTGAGGTCCAAAATCACC
*ul5* qPCR primer R	IDT	5′-CGACCCATCAACACCATCTT
*ul30* qPCR primer F	IDT	5′- GCGAAAAGACGTTCACCAAG
*ul30* qPCR primer R	IDT	5′-CGGAGACGGTATCGTCGTAA
*GAPDH* qPCR primer F		5′-TTCGACAGTCAGCCGCATCTTCTT
*GAPDH* qPCR primer R		5′-CAGGCGCCCAATACGACCAAATC
MISSION(R) siRNA Universal Negative Control #1	Sigma Aldrich	Proprietary
MISSION(R) siRNA Universal Negative Control #1	Sigma Aldrich	Proprietary
MISSION(R) siRNA targeting human CDK2	Sigma Aldrich	Proprietary

**Table 2.** Biological resources

**Bacterial and virus strains**		
One Shot™ TOP10 Chemically Competent *E. coli*	Invitrogen	C404010
*ICP0-RF* HSV-1	Gift from Dr. Bernard Roizman of University of Chicago (Chicago, IL, USA) and Dr. Saul Silverstein of Columbia University (New York, NY, USA).	N/A
*RFP-VP26* HSV-1	Gift from Dr. Lynn Enquist of Princeton University (Princeton, NJ, USA).	N/A
**Experimental models: cell lines**		
Primary human foreskin fibroblasts (HFFs)	American Type Culture Collection (ATCC)	CRL-1634
HEK293T-STING	Generated in Diner *et al.*, 2016 (14)	N/A
U-2 OS	American Type Culture Collection (ATCC)	HTB-96

**Table 3.** Programs, softwares, algorithms

**Software and algorithms**		
Skyline	https://skyline.ms	MacLean et al., 2010 (79)
GraphPad Prism v9	GraphPad, https://www.graphpad.com/scientific-software/prism/	N/A
ImageJ	National Institutes of Health	N/A
NIS Elements AR Analysis	Nikon	N/A
IUPred3	https://iupred3.elte.hu/	Erdős et al., 2021 (46)
GPS 5.0	http://gps.biocuckoo.cn	Wang et al., 2020 (80)
PPSP	http://ppsp.biocuckoo.org/results.php	Xue et al., 2006 (81)
PhosphoPICK	http://bioinf.scmb.uq.edu.au/phosphopick/phosphopick	Patrick et al., 2016 (82)
ELM	http://elm.eu.org	Kumar et al., 2022 (83)
NetPhorest 2.1	http://netphorest.science/index.shtml	Horn et al., 2014 (84)

### Statistical analyses

All PRM data was loaded into and analyzed in Skyline (MacCoss Lab, University of Washington).

All heatmaps, graphs, and statistical analyses were done in GraphPad Prism 9. Throughout this study, * signifies *P* ≤ 0.05, ** *P* ≤ 0.01 and *** *P* ≤ 0.001, as determined by the significance tests indicated in figure legends. For all mass spectrometry (MS), molecular virology, and microscopy data in this manuscript, the quantification workflows, software, replicates (*N* values), results, and graphical display keys can be found in the figure legends and/or experiment-specific descriptions are included in the accompanying Methods section.

### Experimental model and subject details

#### Cell culture, virus strains, and infection protocols

Infection and transfection experiments were performed in HFF human fibroblast cells (ATCC, CRL-1634) or HEK293T-STING cells (generated in Diner *et al.*, 2016 (14)). Virus plaque assays were performed in U-2 OS cells (ATCC, HTB-96).

Cells were grown as monolayers in Dulbecco's modified Eagle's medium (DMEM, high glucose) supplemented with 10% fetal bovine serum (FBS) and 1% penicillin/streptomycin at 37°C in a 5% CO_2_ atmosphere.

The *ICP0-RF* HSV-1 strain used in this study is a gift from Dr. Bernard Roizman of University of Chicago (Chicago, IL, USA) and Dr. Saul Silverstein of Columbia University (New York, NY, USA). The RFP-VP26 HSV-1 strain used is a gift from Dr. Lynn Enquist of Princeton University (Princeton, NJ, USA). Working stocks of the viruses were generated by infecting U-2 OS cells at low multiplicities of infection (MOI = 0.1) and incubated at 37°C until 100% cytopathic effect (CPE) was observed (2–3 days). Both culture supernatants and cells were collected and buffered with MNT buffer (200 mM MES, 30 mM Tris–HCl, 100 mM NaCl, pH 7.4). Supernatants were laid over 10% Ficoll in MNT buffer and subjected to ultracentrifugation (20,000 rpm, 2 h, 4°C with SW28 swinging bucket rotor [Beckman Coulter]) to concentrate virus. Cell-associated virus was collected by sonication and pooled with pelleted cell-free virus. Virus stock titers were determined by plaque assay on U-2 OS monolayers. Virus stocks were stored at −80°C until use.

For all infections in this study, cells were grown to 90–100% confluency prior to infection, and inoculations were performed in a quarter of the standard media volume. Cells were infected for 1 h in 2% FBS + DMEM, rinsed in warm PBS, and returned to 10% FBS + DMEM until sample collection. The multiplicity of infection (MOI) varied by experiment, as indicated in the text or figures.

### Method details

#### Plasmid construction

All IFI16-GFP mutant constructs were generated by a site-directed mutagenesis method in the pEGFP-N1 plasmid for transient expression, pBAD plasmid for protein expression and purification, and LentiORF pLEX-MCS for stable expression in HFFs, as described. ΔIDR-IFI16-GFP, FUS-IFI16-GFP and MBP-IFI16-GFP protein constructs were generated using the In-Fusion® HD Cloning Kit (Takara Bio USA, Inc.) following the manufacturer's instructions.

Plasmids for expression in *Ciona* embryos were generated using the Sox1/2/3 regulatory region backbone (34) IFI16 cassettes were amplified by PCR using proof reading polymerases (Primestar, Takara) and assembled using NEB HiFi DNA assembly according to the manufacturer's instructions.

#### Cell line construction, transfections for expression and knockdown, and lentivirus

IFI16 CRISPR-Cas9 KO cell lines were generated as described previously (35).

Lentiviruses were generated in and harvested from HEK293T cells: packaging vectors psPAX2 and pMD2.G (VSV-G) were co-transfected with a lentiviral transfer vector in a ratio of 1.75:1:2.25 (psPAX2 : pMD2.G : transfer vector) using XtremeGENE HP transfection reagent (Roche Diagnostics) at a ratio of 1:3 (μg DNA : μl XtremeGENE HP). Lentivirus was collected at 48 and 72 h post-transfection in 20% FBS DMEM. Supernatants containing lentivirus were laid onto a 20% sucrose cushion and subjected to ultracentrifugation (25,000 rpm, 2 h, 4°C with SW28 swinging bucket rotor [Beckman Coulter]). Lentiviral pellets were solubilized in freezing buffer (5% Sucrose, 50 mM Tris–HCl, pH 7.4, 20 mM MgCl_2_) and flash-frozen in liquid nitrogen and stored at −80°C until use.

For plasmid transfections, HFF cells were seeded at 2 × 10^5^ cells/ml the evening before transfection with 3 μg of DNA and a 1:3 ratio with X-tremeGENE transfection reagent in Opti-MEM. HEK293T cells were seeded at 70% confluency the day before transfection with 2 μg of DNA instead. Cells were allowed to recover for 24 h before infection.

siRNA oligos were purchased through Sigma Aldrich (custom siRNA consisting of heterogeneous mixture of siRNA that all target the same mRNA sequence). For siRNA-mediated knockdown, HFF cells were seeded at 50,000 to 65,000 cells/ml prior to KD. 20 pmol of siRNA oligos were used per 1 ml well of cells. siRNA was incubated in Lipofectamine RNAiMAX (ThermoFisher Scientific) and Opti-MEM for 5 min, according to the manufacturer's instructions, before being added to cells in fresh, complete DMEM. KD cells were left for 48 h before further experiments and were not passaged during siRNA transfections.

#### RNA isolation and quantitative RT-PCR

Total cellular RNA was purified with the RNeasy Mini kit (Qiagen) following the manufacturer's instructions from 3 × 10^5^ HFF cells. Contaminating DNA was digested with DNase I (Invitrogen) for 15 min at room temperature. RNA was reverse transcribed using the SuperScript IV First-Strand Synthesis Kit (ThermoFisher Scientific) following manufacturer's instructions. Gene-specific primers and the SYBR green PCR master mix (Life Technologies) were used to quantify the resulting cDNA by qPCR on ViiA 7 real-time PCR systems (Applied Biosystems). Relative mRNA quantities were determined using the ΔΔCT method with GAPDH as an internal control.

#### Virus progeny virion titers

Virus titers were determined by plaque assay on U-2 OS monolayers. Cells were lysed by freezing, and both cell-associated and cell-free viruses were collected, briefly sonicated, and serially diluted. Infections were as above, but after 1 h, the inoculum was replaced with 1% METHOCEL (w/v) in DMEM with 10% FBS. After 72 h, when plaques were evident, the cells were incubated with crystal violet [1% crystal violet (w/v) in 50% methanol (v/v)] for 15 min at room temperature before rinsing and plaque quantification.

#### Protein expression and purification of MBP-IFI16-GFP

WT MBP-IFI16-GFP or MBP-IFI16(6A or 6D)-GFP were expressed and purified from Escherichia coli. One Shot™ TOP10 Chemically Competent *E. coli* (Invitrogen) was used for transformation of codon-optimized plasmid encoding His6-MBP-IFI16-GFP and its mutants. Bacteria were cultured until OD_600_ ∼0.8 and induced with 0.5% l-(+)-Arabinose (Sigma-Aldrich, Inc.) at 16°C for 12 h. Bacteria were pelleted at 4,600 × g for 20 min, resuspended in lysis buffer (50 mM Tris–HCl pH 8.0, 150 mM NaCl, 5% glycerol, and Halt™ Protease and Phosphatase Inhibitor Cocktails [Thermo Fisher Scientific]), and lysed on EmulsiFlex C3 (Avestin). Crude lysates were clarified by centrifugation at 35,000 × g for 30 min at 4°C. Clarified lysates were affinity-purified using Ni-NTA Superflow beads (Qiagen), washed six times with wash buffer (50 mM Tris–HCl pH 8.0, 150 mM NaCl, 40 mM imidazole, 5% glycerol), and eluted with 50 mM Tris–HCl pH 8.0, 150 mM NaCl, 250 mM imidazole, 5% glycerol. Eluted proteins were immediately buffer exchanged into 50 mM Tris–HCl pH 8.0, 500 mM NaCl, 5% glycerol using Amicon Ultra-4 Centrifugal Filter Unit with 30k MW filter (MilliporeSigma) and stored at 4°C. Proteins were then subjected to size-exclusion chromatography using a Superdex S200 column (GE Healthcare) in 50 mM Tris–HCl pH 8.0, 500 mM NaCl, 5% glycerol. Fractions were collected, stored at 4°C and only concentrated immediately before *in vitro* assays. All proteins were purified to ≥95% purity and concentrations were determined using Pierce™ BCA Protein Assay Kit (Thermo Fisher Scientific).

MBP-IFI16 was purified in a similar manner and labeled by using Alexa Fluor™ 488 Protein Labeling Kit (ThermoFisher) following manufacturer's manual. The estimated degree of labeling was 0.7 mol of Alexa Fluor 488 per mol of IFI16.

#### In vitro phase separation assay

Assay was performed in CultureWell™ Chambered Coverglass (Invitrogen) coated with 20 mg/ml BSA. Mixtures containing indicated components except for MBP-IFI16-GFP were initially added and incubated in 50 mM Tris–HCl pH 8.0, 5% glycerol and 8% Ficoll. Phase separation was achieved by adding MBP-IFI16-GFP (in 50 mM Tris–HCl pH 8.0, 500 mM NaCl, 5% glycerol) to the mixtures such that the final concentration of NaCl was 83.3 mM.

#### In vitro kinase assay

4 μg of purified MBP-IFI16-GFP was incubated with 1 μl of indicated purified recombinant kinase in 1X kinase reaction buffer (25 mM Tris–HCl pH 7.5, 5 mM beta-glycerophosphate, 2 mM DTT, 0.1 mM sodium orthovanadate, 10 mM MgCl_2_), 10 mM ATP and 2 μM Cy5-DNA. The samples were incubated at 30°C with shaking at 300 rpm. The reaction was then stopped by the addition of 4XTES (4% sodium dodecyl sulfate, 20 mM EDTA, 40 mM Tris–HCl pH 7.4) to 1XTES and frozen at −20°C before sample preparation for mass spectrometry.

#### In vitro transcription assay

HiScribe^TM^ T7 High Yield RNA Synthesis Kit (NEB) was used following manufacturer's manual. Specifically, 100 ng of FLuC positive control template provided by the kit was used for each reaction. 5 μM of purified IFI16 or BSA was added to the reaction mix (to a total of 30 μl) and incubated at 37°C for 4 h. 2 μl of DNase I (NEB) was added to the reaction mix and incubated at 37°C for 15 min. Transcribed RNA was isolated by adding 25 μl of LiCl solution from the kit, incubated at −20°C for 30 min and centrifuged at 15,000 × g, 4°C for 15 min. Pellet was washed with 500 μl of 70% ethanol and centrifuged at 15,000 × g, 4°C for 15 min before being resuspended in 30 μl of water. RNA concentrations were measured using nanodrop.

#### Fluorescence recovery after photobleaching

FRAP was performed using a Nikon A1 confocal microscope equipped with a full incubation chamber maintained at 37°C and supplied with 5% CO_2_. The point region was bleached for 2 s at 100% of maximum laser power of a 405 nm laser. The recovery was recorded at 100 ms intervals for 2 s, and 1 s intervals for 5 s. Images were analyzed in Fiji. Fluorescence intensities of regions of interest (ROIs) were normalized to pre-bleached intensities of the ROIs. The exponential curve was generated by plotting the normalized fluorescence intensity values to time by GraphPad Prism.

#### Fluorescence microscopy and analysis

For imaging of HFF cells, cells were seeded onto sterile 10.5 × 22 mm glass coverslips (Thermo Fisher Scientific) at 2 × 10^5^ cells/ml and infected and treated as indicated. Samples were fixed in 4% paraformaldehyde for 15 min at room temperature and washed three times with PBS. Cells were permeabilized in 0.1% Triton X-100 in PBS for 15 min at room temperature and washed three times with PBS with 0.2% Tween 20 (PBST). Cells were then blocked with 2% bovine serum albumin (BSA), 2.5% human serum in PBST and incubated with primary antibodies for 1 hr (IFI16: Sigma, 1:250; ICP4: Santa Cruz, 1:250 in PBST). Samples were washed three times with PBST and incubated with Alexa Fluor-conjugated secondary antibodies (1:2,000 in PBST). Samples were then washed three times with PBST and incubated with 4′,6-diamidino-2-phenylindole (DAPI) (1:100 in PBS; Thermo Fisher Scientific), washed three times with PBST and mounted using ProLong™ Diamond Antifade Mountant (Thermo Fisher Scientific).

For imaging of *in vitro* phase separation, the chambered coverglass was maintained at 30°C if TEV protease was added using an environmental control chamber, and later lowered to 25°C after 1 h of incubation.

Imaging experiments were done in the Princeton Confocal Imaging Core using a Nikon A1 confocal microscope or an inverted fluorescence confocal microscope (Nikon Ti-E) equipped with a Yokogawa spinning disc (CSU-21) and digital CMOS camera (Hamamatsu ORCA-Flash TuCam) using a Nikon 100X Plan Apo objective with a 100X magnification or 60X magnification with Nikon 60X Plan Apo objective as indicated. To image cells expressing IFI16-GFP during infection with RFP-tagged VP26 HSV-1, a Nikon Ti-E2 confocal microscopy equipped with a super-resolution CSU-W1 SoRa module was used with a Nikon 60X Plan Apo objective. These images were processed with Nikon 3D deconvolution in NIS-Elements AR. Image analysis was performed using ImageJ.

#### Ciona electroporation and imaging

*Ciona intestinalis* adults were supplied from M-REP (San Diego, CA) and embryos were handled using standard procedures as previously described (34). Plasmids were electroporated into embryos in cuvettes containing 800 μl Mannitol seawater according to standard conditions (36). 80 μg of IFI16-GFP containing plasmids were electroporated and 20 μg of H2b::mCh or PH::mAp plasmids were electroporated. Imaging was performed using a Zeiss 880 Confocal with an Airyscan detector in fast mode using a 63×/1.40 Plan-apochromat objective as previously described (34).

#### Nuclear periphery and core fractionation

The protocol was modified from (37). About 1.5 × 10^7^ HFF cells were used per fractionation. Nuclei of the cells were isolated using NE-PER™ Nuclear and Cytoplasmic Extraction Reagents (Thermo Fisher Scientific) following manufacturer's instructions. Isolated nuclei pellets were washed twice with PBS and resuspended in 10 mM Tris–HCl pH 7.4, 1.5 mM KCl, 0.5% Triton X-100, 0.5% Deoxycholate, 2.5 mM MgCl2, with fresh 0.2 M LiCl and protease inhibitors (ratio 1:2 v/v). The mixture was rotated for 1 h at 4°C and centrifuged at 2,000 × g for 5 min at 4°C. Supernatant was further centrifuged at 10,000 × g for 10 min at 4°C to obtain perinuclear fraction (PNF) from the supernatant. Pellet fraction was resuspended in 0.34 M sucrose and centrifuged at 2,000 × g for 10 min at 4°C. The pelleted fraction was dissolved in 8 M urea, sonicated and centrifuged at 10,000 × g for 10 min at 4°C. Supernatant from the latest centrifugation was collected as the core nuclear fraction (CNF). BCA assay was performed to equilibrate protein concentrations between PNF and CNF prior to sample preparation for mass spectrometry analysis.

#### TUNEL assay

Each TUNEL experiment included three biological replicates for each condition and the following controls: (i) cells treated with DNase (10 min) for a positive control and (ii) cells for a negative control (not treated with TUNEL enzyme). A Roche *In Situ* Cell Death Detection Kit (Sigma Aldrich) was used to detect apoptotic cells in 96-well plates. In short, cells were fixed in 4% paraformaldehyde (PFA) for 10 min at room temperature, washed in PBS, and permeabilized with 0.1% TritonX + 0.1% sodium citrate in PBS for 2 min at 4°C. All wells were washed in PBS, and then positive control wells were treated with 10 units of DNase I recombinant + 1 mg/ml BSA + 1 M Tris–HCl pH 7.5 in water for 10 min at room temperature. All wells were washed in PBS again, and then incubated for 1 h at 37°C in TUNEL mix (enzyme solution + label solution). Cells were stained with DAPI and imaged with a Perkin Elmer Operetta automated microscope, scoring apoptotic cells by those positive for TUNEL staining.

#### Immunoaffinity purifications

Cells were washed once with cold PBS and scraped for collection. After pelleting by centrifugation at 300 × g, cells were lysed in lysis buffer (20 mM K-HEPES, pH 7.4, 0.11 M KOAc, 0.1% Tween-20 (v/v), 200 mM NaCl, 1% Triton X-100, 1× PIC/PhIC, 100 U/ml Benzonase [Pierce]). Cells were kept on ice for 30 min with vortexing every 10 min and centrifuged at 8000 × g for 10 min at 4°C. 25 uL GFP-Trap_MA GFP antibody-coupled magnetic beads (Chromotek) was added to each sample and incubated for 1 h at 4°C. The beads were washed three times with wash buffer (20 mM K-HEPES, pH 7.4, 0.11 M KOAc, 0.1% Tween-20 (v/v), 200 mM NaCl, 0.6% Triton X-100). Proteins were eluted in 1× TES (1% sodium dodecyl sulfate, 5 mM EDTA, 10 mM Tris–HCl 7.4) by incubating at 70°C for 10 min then vigorously vortexing for 10 min. Samples were then frozen at −20°C or immediately prepared for mass spectrometry analysis.

#### Chromatin-immunoaffinity purifications

Upon infection of HFF cells with *ICP0-RF* HSV-1 (2 hpi and MOI of 1), 4 × 10^6^ cells per biological replicate were treated with 1% PFA in complete medium (DMEM supplemented with 10% FBS and 1% penicillin/streptomycin) for 7 min at 37°C. Cold glycine was added to a final concentration of 125 mM and incubated at room temperature for 5 min. Cells were washed twice in cold PBS and scraped in PBS supplemented with 1× Halt protease and phosphatase inhibitor cocktail (P/PhIC; Thermo Fisher Scientific). Cells were pelleted and lysed in 50  mM HEPES–KOH, 140 mM NaCl, 10% glycerol, 0.5% NP-40, 0.25% Triton X-100, 1 mM EDTA, and 1:100 P/PhIC to isolate nuclei. Nuclei were pelleted upon centrifugation at  1,350 × g for 10 min at 4°C, subsequently washed in 10 mM Tris–HCl (pH 8.0), 200 mM NaCl, 1 mM EDTA, 0.5 mM EGTA, and 1:100 P/PhIC, and lysed in 10 mM Tris–HCl (pH 8.0), 100 mM NaCl, 1 mM EDTA, 0.5 mM EGTA, 0.1% sodium deoxycholate, 0.5% N-lauroylsarcosine, and 1:100 P/PhIC. Samples were then individually sonicated in a cup horn sonicator (20 sec continuously, medium power, repeated 9 times) with 1 min incubation on ice in between rounds. Samples were brought to 1% Triton X-100 and centrifuged at  20,000 × g, for 10 min at 4°C. IPs were performed on the soluble fractions using 25 μl GFP-Trap_MA GFP antibody-coupled magnetic beads (Chromotek) per IP and incubated for 1 h at 4°C with end-over-end rotation. Beads were washed five times with 50 mM HEPES–KOH (pH 7.6), 100 mM LiCl, 1 mM EDTA, 1% NP-40, and 0.7% sodium deoxycholate, washed once with 10 mM Tris–HCl (pH 8.0), 100 mM EDTA, 50 mM NaCl, and eluted in 50 mM Tris–HCl (pH 8.0), 1% SDS, 10 mM EDTA upon heating at 65°C in a ThermoMixer (Eppendorf) with shaking at 300 rpm for 30 min. Cross-links were reversed in input samples and eluates by incubating at 65°C overnight. All samples were treated with 0.4 mg/ml RNase A and incubated at 37°C for 30 min, and 0.4 mg/ml Proteinase K at 55°C for 2 h. Relative levels of viral genomic DNA were determined by qPCR using the ΔΔCT method with GAPDH as an internal control.

#### Sample preparation for mass spectrometry

Samples were first reduced and alkylated in 5 mM TCEP, 15 mM chloroacetamide for 20 min at 70°C, then acidified to 1.2% phosphoric acid prior to protein extraction. Digestion was performed with trypsin (1:25 of trypsin to sample protein) and suspension trapping columns (S-Trap, Protifi) for 1 h at 47°C, according to the manufacturer's instructions. Following digestion, peptide eluates were resuspended in 10 μl of 1% FA, 1% ACN at a concentration of 1 μg/μl prior to loading on the instrument.

#### Mass spectrometry acquisition

For samples collected in PNF/CNF fractionation and IFI16-GFP IP-PRM:

Peptides were analyzed by nano-liquid chromatography coupled to tandem mass spectrometry with a Q Exactive HF Hybrid Quadrupole-Orbitrap instrument (Thermo Scientific) using data-dependent acquisition (DDA). For DDA, Peptides (2 μl injections) were separated with a linear gradient of 3% solvent B to 30% solvent B gradient (solvent A: 0.1% FA, solvent B: 0.1% FA, 97% ACN) over 150 min at a flow rate of 250 nl/min on a self-packed 50 cm Reprosil C18 column (Dr. Maisch GmBH) or with an EASYSpray C18 column (75 μm × 50 cm, Thermo) heated to 50°C. The full scan range was set to 350–1,800 *m*/*z* at 120,000 resolution and recorded in profile. The top 15 most intense precursors were subjected to DDA HCD fragmentation with a normalized collision energy (NCE) of 28. Tandem MS/MS spectra were acquired at a resolution of 30,000 and an automatic gain control (AGC) target set to 1e5 and a 150 ms maximum injection time (MIT). Precursors were selected with an isolation window of 1.2 *m*/*z*. For PRM analyses, peptides were separated chromatographically identically to the DDA method. A full MS survey scan was acquired at resolution of 30,000, an AGC target of 3e6, MIT of 15 ms, scan range of 350–1,800 *m/z*, and data was collected in profile. For the PRM scans, the resolution was set to 30,000, an AGC target of 1e5, a MIT of 60 ms, loop count of 20, precursors were selected with a 0.8 *m*/*z* isolation window, a fixed first mass of 125 *m/z*, NCE of 27, and spectra were collected in profile.

For samples collected in *in vitro* kinase assays:

Peptides from samples with kinase added were pooled to make a master mix and subjected to DDA analysis. Peptides (2 μl injections) were separated with a linear gradient of 3% solvent B to 30% solvent B gradient (solvent A: 0.1% FA, solvent B: 0.1% FA, 97% ACN) over 60 min at a flow rate of 250 nl/min on a self packed 50 cm Reprosil C18 column (Dr Maisch GmBH) heated to 50°C. The full scan range was set to 350–1500 *m*/*z* at 120,000 resolution and recorded in profile. The top 20 most intense precursors were subjected to DDA HCD fragmentation with a normalized collision energy (NCE) of 28. Tandem MS/MS spectra were acquired at a resolution of 15,000 and an automatic gain control (AGC) target set to 1e5 and a 25 ms maximum injection time (MIT). Precursors were selected with an isolation window of 1.2 *m*/*z*. For PRM analyses, peptides were separated chromatographically identically to the DDA method. A full MS survey scan was acquired at resolution of 120,000, an AGC target of 3e6, MIT of 200 ms, scan range of 150–2,000 *m/z*, and data was collected in profile. For the PRM scans, the resolution was set to 15,000, an AGC target of 2e5, a MIT of 200 ms, loop count of 25, precursors were selected with a 1.2 m/z isolation window, a fixed first mass of 125 *m/z*, NCE of 27, and spectra were collected in profile.

#### PRM library and analysis of MS data

PRM assays were designed and analyzed using the Skyline Daily software. Dominant proteotypic peptides for each IFI16 phosphorylation site were selected based on detection levels in DDA and the elution and fragmentation profiles were experimentally determined for each targeted peptide. Peptide abundance was quantified using the summed area under the curve of 3–5 fragment ions per peptide. Peptide abundance values were normalized by the abundance value of a selected unmodifiable peptide of IFI16 and scaled to the mock condition in each IP. Two-tailed Student's *t*-tests were performed using GraphPad Prism 9.

Tandem MS spectra collected from DDA mode were analyzed by Proteome Discoverer v2.4 (Thermo Fisher Scientific). For identification of protein enriched in PNF, abundance ratio for each protein was calculated by taking the ratio of PNF/CNF of average abundance values across biological replicates, and two-tailed Student's *t*-tests were performed to calculate the *P*-value for the abundance ratio.

## RESULTS

### IFI16 undergoes liquid–liquid phase separation *in vitro*

We previously observed that IFI16 undergoes dynamic changes in its subnuclear localization at the early stages (30 min–2 h) of HSV-1 and HCMV infections (14). IFI16 leaves the nucleolus and forms discrete foci at sites of viral genome deposition at the nuclear periphery (14). The IFI16 foci have a dynamic behavior, forming round puncta that are uniform in size and that appear and disappear on the order of minutes (Supplementary Figure S1A, Movie 1). The initial enrichment of IFI16 within nucleoli, which are phase-separated organelles, and its dynamic spherical puncta formation led us to ask whether IFI16 undergoes liquid–liquid phase separation (LLPS) during the initial stages of binding to incoming viral DNA. LLPS is a process involving the formation of membrane-less compartments mediated by weak interactions among the components, and has been shown to be involved in regulating intracellular organization, signaling transduction, transcription, and cell stress response (38–41).

**Figure 1.**
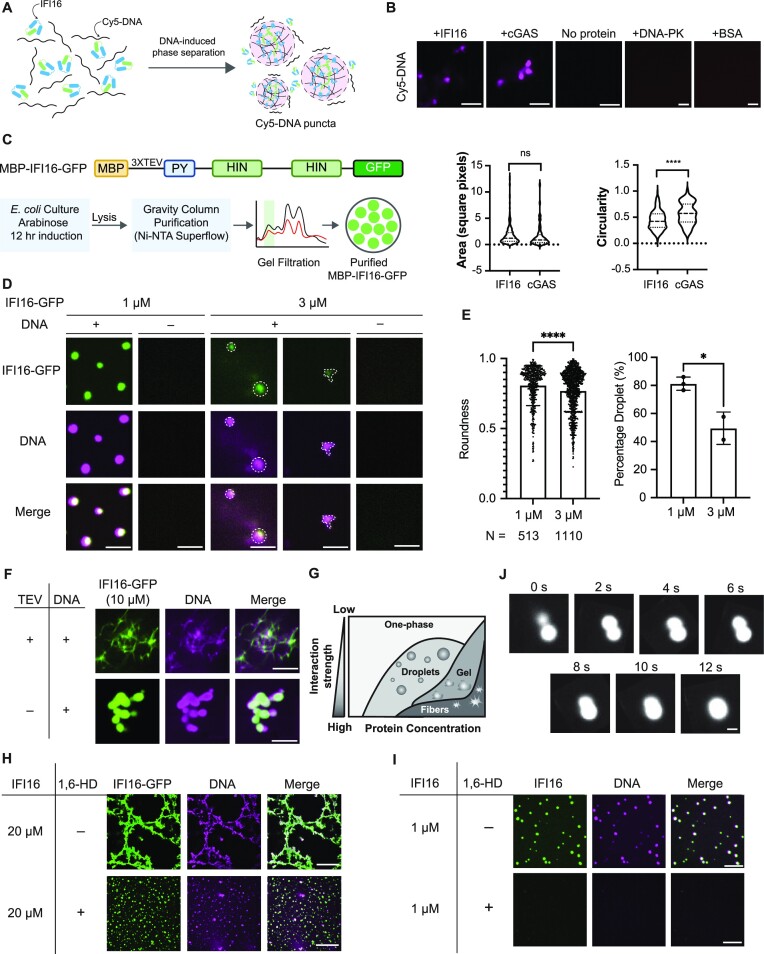
IFI16 forms droplets and filaments mediated by LLPS *in vitro* in the presence of DNA. (**A**) Schematics of IFI16-DNA interactions leading to LLPS. Green and blue boxes represent the pyrin domain and HIN domains of IFI16 respectively. (**B**) Images of 5 μM Cy5-DNA in the presence or absence of purified 5 μM cGAS or IFI16. Areas and circularities of Cy5-DNA puncta were quantified using Fiji. Scale bar, 5 μm. Statistical analysis was performed using unpaired t-test. (**C**) Construct of MBP-IFI16-GFP and schematics of the optimized protocol for purifying MBP-IFI16-GFP. MBP, maltose-binding protein. PY, pyrin domain. Monomeric eGFP was used. (**D**) Representative images of IFI16-GFP LLPS at 1 or 3 μM incubated with equimolar Cy5-DNA after TEV protease treatment. Scale bars, 1 μm. (**E**) Quantitative measurements of roundness and percentage of droplets of IFI16-GFP at 3 and 1 μM. Percentage droplets at each concentration was calculated as the percentage of puncta with roundness > 0.8. Roundness values were calculated using Fiji. Statistical analysis was performed using unpaired t-test. Values are means ± SEMs (*n* = 3). (**F**) Representative images of 10 μM IFI16-GFP or MBP-IFI16-GFP incubated with equimolar Cy5-DNA in the presence or absence of TEV protease. Scale bar, 5 μm. (**G**) Schematic phase diagram representing distinct material phases that can be undertaken by LLPS proteins at varying concentrations and interaction strength amongst constituent molecules. (**H**) Representative images and quantification of IFI16-GFP LLPS at 20 μM in the presence or absence of 10% 1,6-HD. Scale bar, 5 μm. (**I**) Representative images of untagged IFI16 labeled by Alexa Fluor 488 LLPS at 1 μM in the presence or absence of 10% 1,6-HD. Scale bar, 5 μm. (**J**) Time-lapse micrographs of merging droplets of IFI16-GFP (20 μM) in the presence of 10% 1,6-HD and Cy5-DNA. Scale bar, 1 μm.

We first determined whether IFI16 alone is sufficient to cluster DNA *in vitro* (Figure 1A). Purified IFI16 (10 μM) was mixed with equimolar amounts of Cy5-labelled vaccinia virus dsDNA 70mer (VACV 70mer). Cyclic GMP-AMP synthase (cGAS), a DNA sensor known to undergo LLPS in the cytoplasm (41), was included as a positive control. Confocal microscopy showed robust Cy5-DNA puncta formation in the presence of either IFI16 or cGAS (Figure 1B), suggesting that either IFI16 or cGAS is sufficient to cluster DNA *in vitro*. As negative controls, we incubated Cy5-DNA with either BSA, which does not bind to DNA, or DNA-PK, which binds to DNA but has not been shown to undergo LLPS. Both proteins failed to induce Cy5-DNA puncta (Figure 1B). Although we did not observe a difference in the size of the Cy5-DNA puncta in the presence of cGAS or IFI16, we noticed that DNA puncta induced by cGAS appeared as more spherical than those induced by IFI16 (Figure 1B). This may be driven by the difference in the type of oligomerization that cGAS or IFI16 undergo, with cGAS known to dimerize and IFI16 shown to undergo oligomerization that can expand into filaments.

To directly visualize IFI16 morphology upon its binding to DNA *in vitro*, we purified IFI16-GFP with a maltose binding protein (MBP) and 3X TEV protease cleavage sites tagged at the IFI16 N-terminus (Figure 1C). Only in the presence of DNA, MBP-cleaved IFI16-GFP formed micrometer-sized spherical droplets that colocalized with Cy5-DNA (Figure 1D). The IFI16 droplet formation was concentration-dependent, being most apparent at the lowest concentration tested, i.e. 1 μM. At a higher concentration (3 μM), in addition to forming droplets, IFI16-GFP started to expand into filamentous structures (Figure 1D). The prevalence of droplet formation at 1 μM was confirmed by quantification (Figure 1E). Further increasing the IFI16 concentration to 10 μM caused the MBP-cleaved IFI16-GFP to display fully filamentous structures (Figure 1F, first row). Additionally, even at 10 μM, the presence of MBP, which has been reported as a solubility-enhancing tag (42,43), in the uncleaved MBP-IFI16-GFP led to droplet formation that colocalized with Cy5-DNA (Figure 1F, second row and Movie 2). The observed state for the uncleaved IFI16 fits into a model in which the MBP affects the interaction strength among IFI16-GFP molecules, likely by inhibiting PY-dependent homotypic interactions and IFI16 oligomerization. Altogether, these results illustrate that the clustering of IFI16-GFP is dependent on the presence of DNA, and its material state transition from droplets to filaments is linked to its concentration and interaction strength, fitting into the canonical model for proteins capable of undergoing LLPS (Figure 1G) (44).

To further validate the IFI16 LLPS *in vitro*, we incubated low or high concentrations of IFI16-GFP (1 or 20 μM) with Cy5-DNA in the presence or absence of 1,6-hexanediol (1,6-HD), a chemical reported to selectively inhibit liquid-phase condensates (45). For both concentrations tested, IFI16-GFP morphology was significantly altered by the addition of 1,6-HD (Figure 1H-I). At 20 μM, the presence of 1,6-HD, which was added prior to the addition of the TEV protease and the purified IFI16 protein, prevented filament formation, arresting IFI16-GFP in a droplet form (Figure 1H). The observation that filaments are not completely dissolved by 1,6-HD could be attributed to a number of factors, including the primary inhibition of hydrophobic interactions by this treatment (45) and expected maintenance of IFI16-DNA interaction and PY charge-mediated oligomerization. Under the same condition at 1 μM, the IFI16-GFP droplets were abolished (Supplementary Figure S1A). Further supporting the involvement of LLPS in IFI16 droplet formation, we observed that droplets fused rapidly, within seconds (Figure 1J). Altogether, the *in vitro* spherical puncta formation in the presence of DNA, together with the response to 1,6-HD treatment and the observed droplet fusion all point to the ability of IFI16 to undergo LLPS *in vitro*.

To confirm that the C-terminal GFP did not impact the IFI16 LLPS capacity, we purified untagged IFI16, which we then labeled with Alexa Fluor 488. Similar to IFI16-GFP, untagged IFI16 displayed a concentration-dependent LLPS morphology, forming droplets and filaments at similar concentrations (1 and 10 μM respectively, Figure 1I and S1B). The droplets formed by untagged IFI16 were also inhibited by 1,6-HD (Figure 1I). These data suggest that the GFP did not affect the LLPS capacity of IFI16 *in vitro*.

**Figure 2.**
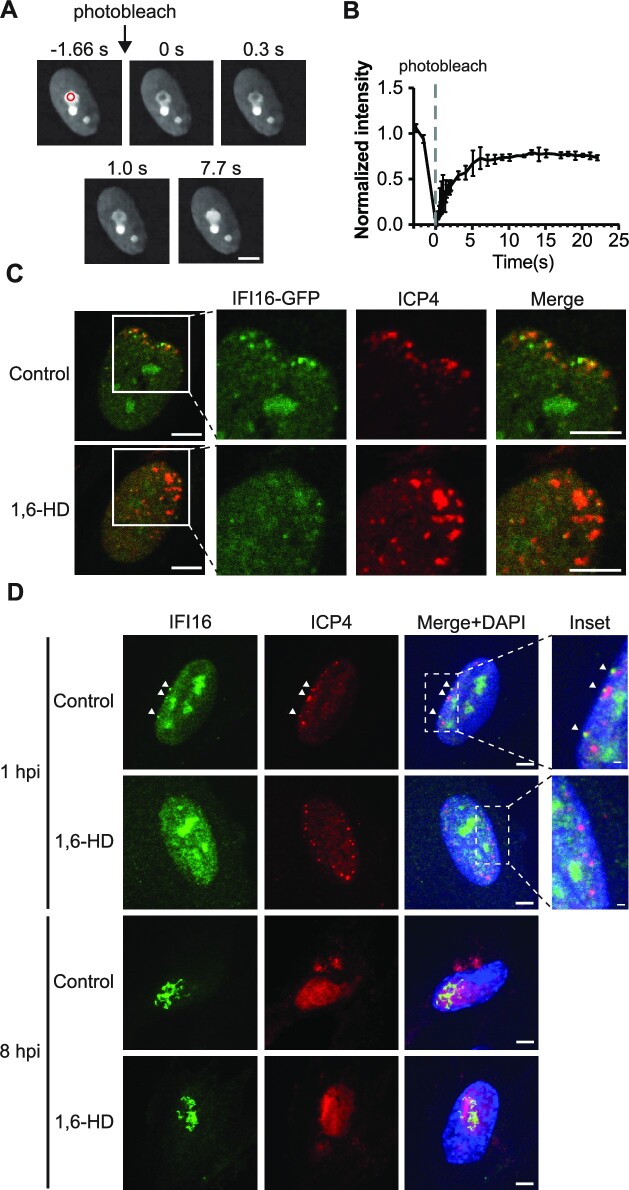
IFI16 displays dynamic exchange with surroundings and IFI16 puncta formation at the nuclear periphery can be inhibited by 1,6-hexanediol. (**A**) FRAP images showing the recovery of IFI16-GFP after photobleaching. HFF cells stably expressing IFI16-GFP was subjected to FRAP analysis. The red circle indicates the area of photobleaching. (**B**) Recovery kinetics of IFI16-GFP in (A). Values are means ± SEMs (*n* = 3). (**C**) IFI16-GFP stably expressing HFFs were infected with *ICP0-RF* HSV-1 (MOI = 10, 1 hpi), treated with DMSO or 300 mM 1,6-HD for 10 min and stained for ICP4. Scale bar, 5 μm. (**D**) Confocal microscopy images showing WT HFFs infected with *ICP0-RF* HSV-1 (MOI = 10, 1 hpi or 8 hpi), treated with DMSO or 300 mM 1,6-HD for 10 min and stained for endogenous IFI16 and ICP4. Scale bar, 5 μm or 1 μm for inset.

We next asked whether IFI16 also displays LLPS behavior in cells. Within the nucleus, IFI16 has been shown to display nucleolar, as well as diffuse nuclear localizations, which may be linked to different cell states and cell types, as well as different epitopes targeted by the antibodies used. To assess a possible IFI16 LLPS behavior in cells, we first monitored uninfected cells where we noticed pronounced nucleolar localization. Using primary human fibroblasts stably expressing IFI16 fused to monomeric eGFP (IFI16-eGFP), we monitored recovery after photobleaching within the nucleolus. IFI16 fluorescent signals recovered rapidly, within seconds, suggesting that nucleolar IFI16 exhibits a highly dynamic exchange with its surroundings (Figure 2A-B). The nucleolar population of IFI16 likely contributes to the formation of perinuclear puncta during infection (Supplementary Figure S1C), as we observed an anti-correlation between the fluorescence intensities of IFI16-eGFP in the nucleolus and those at the nuclear periphery upon infection with HSV-1 (Supplementary Figure S1D). To assess whether LLPS is involved in the recruitment of IFI16 to incoming viral DNA, we tested the impact of 1,6-HD treatment on IFI16 localization during HSV-1 infection. As HSV-1 is known to target IFI16 for degradation (8), we performed infections with an HSV-1 strain that harbors a mutation in the ring finger (RF) domain of the viral E3 ubiquitin ligase ICP0 and thereby lacks the ability to suppress IFI16 functions (*ICP0-RF* HSV-1). As expected, we observed IFI16-GFP puncta localizing at the nuclear periphery, at sites of viral genome deposition, early in infection, i.e. at 1 h post infection (hpi) with *ICP0-RF* HSV-1 (Figure 2C). By 8 hpi, IFI16-GFP displayed a filamentous appearance (Supplementary Figure S1E). In both instances, IFI16 co-localized with the viral immediate-early protein ICP4, which marks sites of viral genomes. These findings were recapitulated when staining for endogenous IFI16 at similar time points, indicating that these phenotypes are not an artifact of the overexpression system (Figure 2D). Treatment with 1,6-HD inhibited the ability of either IFI16-GFP or endogenous IFI16 to become enriched and form puncta at viral genome sites at the nuclear periphery (Figure 2C-D). On the other hand, as expected when considering the metastability of proteins with LLPS properties (44) (Figure 1G), once IFI16 filaments were formed at 8 hpi, 1,6-HD treatment was no longer able to reverse this solid-like state (Figure 2D). These observations confirm that the 1,6-HD treatment specifically inhibits liquid-state condensates. Taken together, these results demonstrate that the puncta formation of IFI16 is mediated by LLPS, and that this condensation ability is required for the localization of IFI16 to viral genomes at the nuclear periphery.

### IFI16 contains an intrinsically disordered region required for LLPS and antiviral function

**Figure 3.**
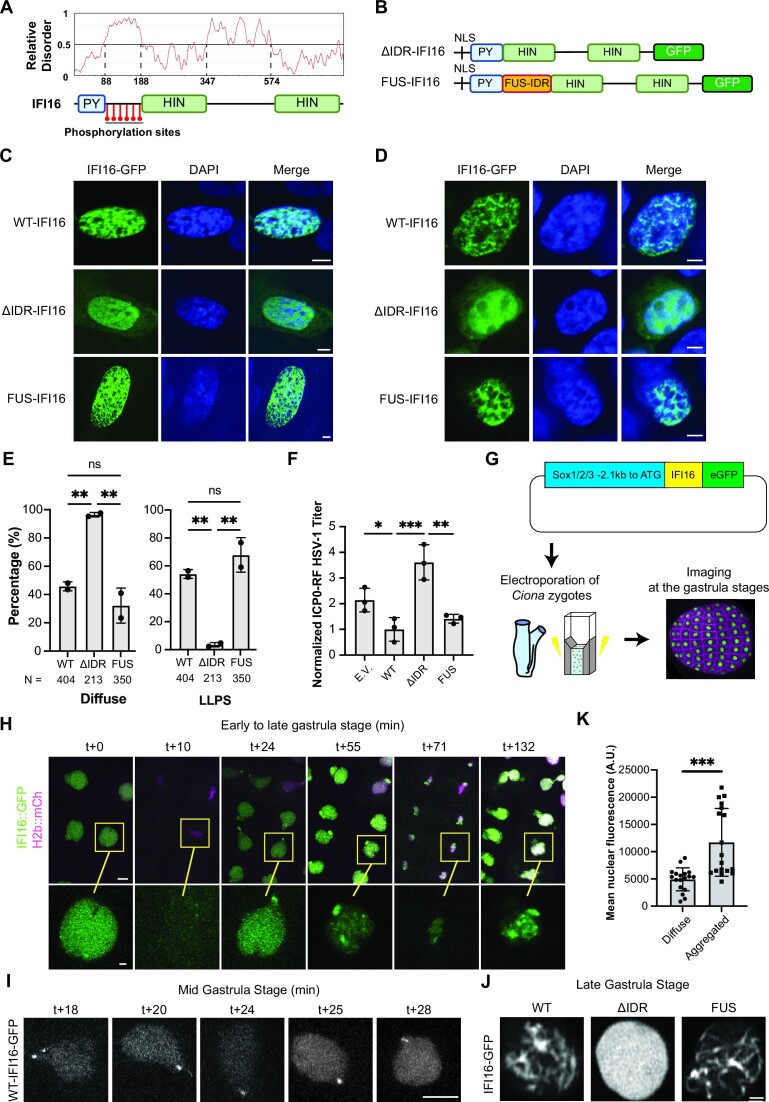
*The intrinsically disordered region (IDR) within IFI16 is necessary for IFI16 LLPS both* *in* *cells and in vivo*. (**A**) Relative disorder score of full length IFI16 as predicted by Prediction of Intrinsically Unstructured Proteins (IUPred3) and phosphorylation sites identified previously using immunoaffinity purification–mass spectrometry. (**B**) Construct schematics of IFI16 mutants. (**C**) Representative images of GFP-tagged WT, ΔIDR-IFI16 or FUS-IFI16 transiently transfected in WT HFFs. Scale bars, 5 μm. (**D**) Representative images of GFP-tagged WT, ΔIDR-IFI16 or FUS-IFI16 transiently transfected in HEK293T-STING cells. Scale bars, 5 μm. (**E**) Quantification of percentage of cells displaying LLPS (including filament or punctate) or diffuse phenotypes of WT or mutant IFI16-GFP in **(C)**. N indicates the number of cells quantified in total. Statistical analysis was performed using unpaired *t*-test. Values are means ± SEMs (*n* = 2). (**F**) Progeny virus titers from HEK293T-STING cells transfected with indicated plasmids and infected with *ICP0-RF* HSV-1 virus (MOI = 0.2). Cell-associated and cell-free virus were pooled at 24 hpi, and the titers of the virus on U-2 OS cells were determined by plaque assay. Values are means ± SEMs (*n* = 3). Statistical analysis was performed using ordinary one-way ANOVA. E.V., empty vector control expressing GFP only. **(G)** Workflow schematic of *Ciona* electroporation. **(H)** Time lapse microscopy (in min) of IFI16::GFP over the course of 2 mitoses in *Ciona* epidermis. Scale bar, 5 μm. (**I**) Maximal projection confocal sections of IFI16::GFP puncta formation in *Ciona* nuclei at mid gastrula stages (in min). Scale bar, 5 μm. (**J**) Confocal sections of single *Ciona* nuclei at late gastrula stages at high magnifications. Scale bar, 1 μm. (**K**) Quantification of nuclear fluorescence intensity of IFI16::GFP in neurula embryos. Statistical analysis was performed using unpaired *t*-test.

Proteins with capabilities to undergo LLPS often contain multivalent domains or intrinsically disordered regions (IDR) to mediate weak and transient interactions (44). To determine if IFI16 contains disordered regions, we performed a bioinformatics analysis using Prediction of Intrinsically Unstructured Proteins (IUPred3) (46). Two regions of disorder were predicted (aa 88–188 and 347–574, Figure 3A). The most evident IDR region, positioned between the PY and first HIN domain, coincides with the region that we previously reported to be decorated by multiple phosphorylation sites (11,35). Accumulating evidences have suggested that phosphorylation events within disordered regions can contribute to either promoting or suppressing LLPS (47,48), with a recent study demonstrating phosphorylation signatures present specifically in either condensates or the soluble populations of phase separating proteins (49). Hence, we focused on characterizing the possible contribution of the first IDR to the observed IFI16 LLPS. We generated IFI16-GFP constructs containing either an IDR deletion (ΔIDR-IFI16) or a replacement of the IFI16 IDR with FUS aa 1–163 (FUS-IFI16) (Figure 3B), an IDR known to be sufficient to induce LLPS (42). Following the equivalent expression of these IFI16-GFP constructs in primary human fibroblasts (HFFs) and HEK293T cells (Supplementary Figure S2A), we quantified the percentage of cells displaying diffuse or LLPS IFI16-GFP phenotypes, either punctate or filamentous (Supplementary Figure S2B). We found that ΔIDR-IFI16 lost the ability to form puncta or filaments, even in cells with high expression levels of this construct, whereas the FUS-IFI16 rescued this ability to the WT level (Figure 3C-E). We next asked if the loss of LLPS impacts the antiviral function of IFI16. For this, we used an IFI16-deficient cell line that expresses STING (HEK293T-STING) and which has been shown to allow the reconstitution of the STING-TBK1-IRF3 signaling axis to assess cellular antiviral responses (14,50). Infection of cells expressing ΔIDR-IFI16 with *ICP0-RF* HSV-1 resulted in three-fold higher virus titers than cells expressing WT-IFI16 and greater than two-fold higher than cells expressing FUS-IFI16, indicating a defect in the ability of ΔIDR-IFI16 to inhibit viral production (Figure 3F). Altogether, these results suggest that the IDR is necessary for promoting IFI16 LLPS and antiviral function.

### IFI16 IDR regulates LLPS *in vivo*

To determine whether the IDR is also relevant in regulating IFI16 aggregation *in vivo*, we took advantage of a recently developed system for observing nuclear proteins in the embryos of *Ciona intestinalis* (34). *Ciona* diverged from modern vertebrates over 500 million years ago and provides an evolutionarily simple model system (51,52). Directly relevant for this study, we selected *Ciona* given reports that demonstrated its ease of use specifically for visualizing the dynamics of phase separating proteins with high spatial and temporal resolution as they transition across different biophysical states (34). Fluorescently-tagged proteins can be readily expressed by electroporation in *Ciona* embryos, and the large nuclei of these embryo enhance the visualization of condensates for nuclear proteins. As *Ciona* does not express a direct sequence homolog of IFI16, it provides the ability to reconstitute and monitor IFI16 material states in an *in vivo* model system. We expressed WT, ΔIDR, or FUS IFI16-GFP in the ectoderm of live, developing *Ciona* embryos under the regulation of a Sox1/2/3 promoter (Figure 3G). The concentration-dependent distributions and dynamics of these IFI16 constructs were visualized in live embryos using time-lapse microscopy (Movie 3). WT IFI16-GFP displayed evident nuclear localization in early gastrula embryos, being distributed uniformly throughout the nucleus (Figure 3H). No IFI16-GFP was retained on the chromatin during mitosis. After the first mitosis, puncta formed, typically starting at the sites closest to the former cleavage furrow. By the mid gastrula stage, IFI16 puncta were consistently visible at the nuclear periphery (Figure 3H and I). These puncta grew into larger nuclear aggregates by the late gastrula stage (approximately two hours and two rounds of mitosis later, Figure 3J). After these aggregates formed, IFI16 was retained on the chromatin during the next mitosis. In contrast, ΔIDR IFI16-GFP retained a diffuse nuclear distribution throughout gastrula stages (Figure 3J and Supplementary Figure S2C), with no aggregation being detected even at late stages (Figure 3J). Similar to our observations in mammalian cells, the addition of a FUS domain rescued the ability of IFI16 to aggregate. FUS-IFI16-GFP aggregates were evident immediately, starting at early gastrula stages (Supplementary Figure S2C). The FUS-IFI16-GFP fibrous aggregates were retained on the chromatin after the first mitosis and were present throughout development (Supplementary Figure S2C, Movie 4). These aggregates had a filamentous appearance, forming multiple string-like patterns. Since expression levels of transgenes in *Ciona* embryos can be variable, we took advantage of this property and measured the fluorescence intensity of IFI16-GFP in neurula stage embryos, comparing nuclei where aggregates were observed with those without aggregates. Nuclei that contained aggregates typically had 2- to 3-fold higher fluorescence intensities (Figure 3K), suggesting that a critical nuclear concentration of IFI16 needs to be reached for aggregates to form. Altogether, consistent with our findings in mammalian cell culture, these results demonstrated that the WT IFI16 forms concentration-dependent puncta and filamentous aggregates *in vivo*, a property that requires the presence of the IDR.

### Multiple phosphorylation sites within the IDR combinatorially regulate IFI16 LLPS

Having established the importance of the IDR in regulating IFI16 LLPS *in vitro* and *in vivo*, we next asked what properties of the IDR facilitate this function. Given that we previously observed that this IFI16 region contains multiple phosphorylation sites (11,35), we asked whether these phosphorylation events (S95, S106, T149, S153, S168, and S174) contribute to the regulation of the IFI16 LLPS. Using sequence alignment, we observed that most of the identified phosphorylation sites within the IDR are conserved among different primate species (Supplementary Figure S3A, B). Next, we assessed whether these IFI16 sites become phosphorylated early in HSV-1 infection, at time points when IFI16 binds to the incoming viral DNA and exerts the dynamic puncta behavior. To accurately define the IFI16 phosphorylation status, we designed a targeted mass spectrometry assay using parallel reaction monitoring (PRM) that detects signature parameters for each of the predicted phosphorylated peptides. This PRM assay was then applied following enrichment of IFI16 via immunoaffinity purification (IPs) at one- and six-hours post infection (hpi) with *ICP0-RF* HSV-1 (Supplementary Figure S4A, Supplementary Table S1). We reliably detected fragment ion peaks for five out of the six candidate phosphorylated peptides (Supplementary Figure S4B), confirming the presence of S95, S106, T149, S153 and S168 phosphorylation sites at both one and six hpi (Supplementary Figure S4C).

**Figure 4.**
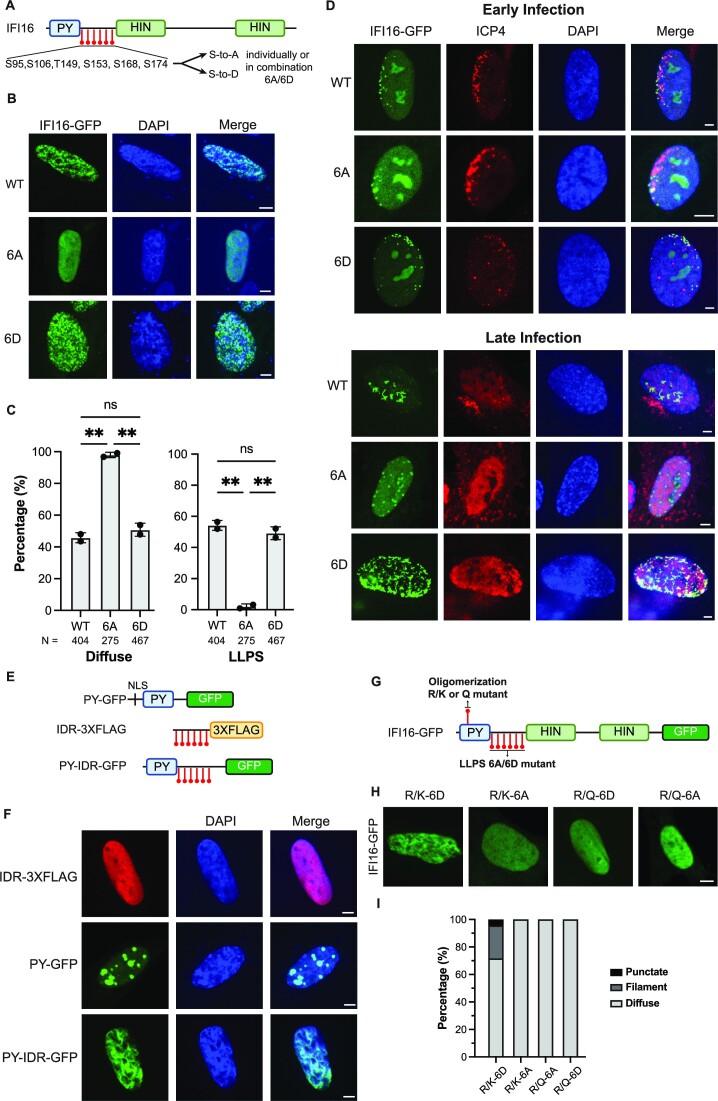
Multiple phosphorylation sites within the IDR combinatorially regulate IFI16 LLPS and antiviral functions. (**A**) IFI16 schematic showing mutated sites within the IDR. (**B**) Representative images of WT, 6A- or 6D-IFI16-GFP transiently expressed in WT HFFs. Scale bar, 5 μm. (**C**) Quantification of percentage of cells (*N* > 115) displaying LLPS (filament or puncta) or diffuse phenotypes in cells expressing WT IFI16 or those described in (B). Statistical analysis was performed using ordinary one-way ANOVA. Values are means ± SEMs (*n* = 2). (**D**) Representative images of stable cell lines expressing either WT, 6A- or 6D-IFI16-GFP in IFI16-KO HFFs at early or late stage of infection. Images were taken at the edge of developing plaques and cells were infected with *ICP0-RF* HSV-1 (MOI 0.1) and fixed at 24 hpi. (**E**) Construct schematics of IFI16 truncation mutants. (**F**) Representative images of IFI16 truncation mutants transiently expressed in WT HFFs. Scale bar, 5 μm. (**G**) Schematic showing the mutated sites within PY and IDR. (**H**) Representative confocal images for cells transiently expressing each of the IFI16 mutants in WT HFFs. Scale bar, 5 μm. (**I**) Quantification of percentage of cells (*N* > 100) displaying punctate, filament or diffuse phenotype in cells transiently expressing each of the IFI16 mutants as described in (H). Quantification is shown for one representative biological replicate.

To determine whether the identified phosphorylation sites impact the IFI16 LLPS capability, we generated a series of single phosphorylation mutants. Each site was individually converted from a serine/threonine to either an alanine or aspartate to introduce an unmodified residue or a phosphorylation mimic, respectively (Figure 4A). In agreement with our *in vivo* observations, filamentous IFI16 phenotypes were correlated with IFI16 expression levels in cells (Supplementary Figure S4D). However, the presence of the single phospho-mutants did not significantly change the ability of IFI16 to form puncta or filaments (Supplementary Figure S4E-F). Additionally, the expression of equivalent levels of the single mutants (Supplementary Figure S4G) did not result in significant differences in virus titers when compared to the WT IFI16 (Supplementary Figure S4H). Hence, we reasoned that a single phosphorylation event is not sufficient to control the LLPS status or antiviral function of IFI16.

To assess whether a combinatorial effect is at play, we generated IFI16 constructs containing all six phosphorylated residues mutated to alanine or aspartate (6A- and 6D-IFI16-GFP, respectively) and expressed these in HFFs (Figure 4A). In the absence of infection, 6A-IFI16-GFP mutant transiently expressed in HFFs lost the ability to form puncta or filaments, whereas the 6D-IFI16-GFP displayed normal or even enhanced puncta and filament formation compared to WT IFI16 (Figure 4B-C). Given these differences, we next investigated these combinatorial phosphomutants during infection. To ensure that the tested impact on IFI16 localization and antiviral functions is the result of the presence of the phosphomutants and not contributed by endogenous IFI16, we generated IFI16 CRISPR/Cas9-mediated knockouts in HFFs and stably expressed CRISPR-resistant forms of WT-IFI16-GFP, 6A-IFI16-GFP or 6D-IFI16-GFP in the knockout background (Supplementary Figure S4I).

Using the stable cell lines expressing the phosphomutants, we examined the impact of phosphorylation on the ability of IFI16 to bind incoming viral genomes at the nuclear periphery during the initial stages of HSV-1 infection. To be able to track virus capsids, we infected the stable cell lines with an HSV-1 strain that expresses the major capsid surface protein, VP26, fused to monomeric red fluorescent protein (*HSV-1::rfp-vp26*). Upon detection of RFP-tagged viral capsids at the outer nuclear periphery, we observed the simultaneous and adjacent association of WT, 6A or 6D IFI16-GFP puncta at the inner nuclear periphery during early infection with *ICP0-RF* HSV-1 at 2 hpi (Supplementary Figure S5A). As an orthogonal approach to confirm that phosphorylation does not impact the capacity of IFI16 to bind viral DNA during infection, we performed a chromatin immunoprecipitation (ChIP) followed by quantitative PCR at 2 hpi, when viral genomes enter the nucleus. We found that both 6A and 6D IFI16 are capable of binding to viral genomes to a similar extent as WT IFI16 (at genomic loci *ul30* and *ul5*, Supplementary Figure S5B). Altogether, these findings indicate that the phosphorylation state of IFI16 does not impact the ability of IFI16 to be recruited to the site of viral genome deposition and bind to viral DNA.

We next investigated whether the localization of IFI16 to viral genomes is sustained throughout infection upon the development of nuclear viral replication compartments. We analyzed cells within developing HSV-1 plaques and assessed IFI16 co-localization with a viral protein marker for viral genomes, ICP4, at both plaque edges (early infection) and plaque interiors (late infection). Early in infection, we observed that both 6A and 6D mutants of IFI16 retain their co-localization with ICP4 at the nuclear periphery. In contrast, later in infection, relative to either WT- or 6D-IFI16 formation of filaments within viral replication compartments, 6A-IFI16 failed to transition from initial puncta to filaments (Figure 4D). These observations suggest that the phosphorylation state of IFI16 impedes its ability to progress through LLPS stages (Figure 1G) to filaments.

Our observations that 6A-IFI16 still forms puncta at the nuclear periphery early in infection, but fails to develop filaments later in infection, led us to propose that the initial puncta formation at the nuclear periphery is facilitated by DNA-binding and PY-mediated oligomerization. These puncta then transition through IDR phosphorylation-regulated phase separation stages as viral replication compartments form, transitioning from droplet-like aggregates to filaments at later stages of infection. To test this hypothesis, we generated IFI16 truncation mutants bearing 1) only the PY domain (PY-GFP), 2) only the IDR domain (IDR-3XFLAG), and 3) both PY and IDR domains (PY-IDR-GFP, as in (14)) (Figure 4E). We observed robust puncta formation for PY-only mutants, suggesting that the PY domain alone is sufficient to give rise to puncta. However, filamentation was only observed in cells expressing PY-IDR-GFP (Figure 4F). These results suggest that both PY and IDR domains are required for IFI16 filamentation, implying a possible synergistic interaction between the two domains.

To investigate the relationship between the IDR LLPS and PY oligomerization functions, we took advantage of our previously identified point mutations that can control the IFI16 PY-mediated oligomerization (24). Specifically, mutating the PY R23 residue to a structural mimic (R23Q), but not to a charge mimic (R23K), inhibited IFI16 oligomerization. Hence, we generated combinatorial IFI16 mutants bearing gain- or loss-of-function mutations within both PY and IDR and tested their abilities to form aggregates in cells by confocal microscopy (Figure 4G). Cells transiently expressing IFI16 that is deficient in either oligomerization or LLPS failed to form filaments. In contrast, IFI16 bearing gain-of-function mutations in both PY and IDR (R/K-6D) rescued the ability to form filaments (Figure 4H-I). This indicates that the oligomerization and LLPS abilities of IFI16 act synergistically to promote its aggregate formation in cells, which is necessary for its antiviral functions.

### IFI16 LLPS-deficient mutant has dampened capacity for cytokine induction but maintained ability to suppress viral gene expression

**Figure 5.**
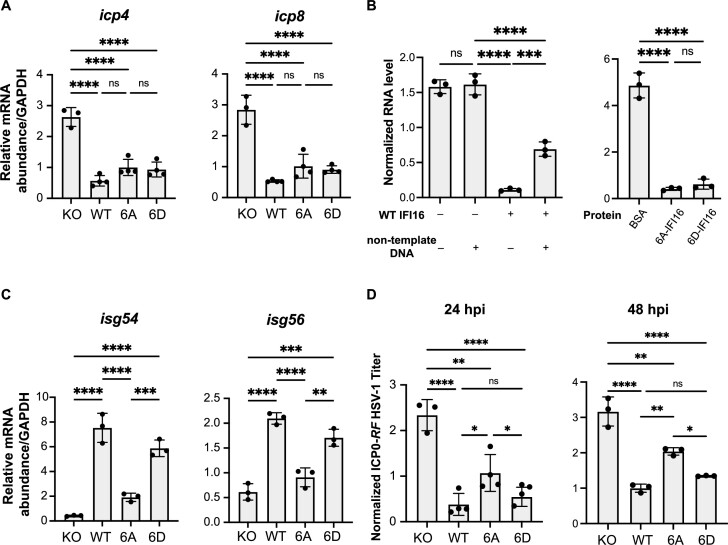
IFI16 phosphorylation does not impact transcriptional suppression and specifically promotes cytokine induction. (**A**) Relative mRNA levels of *icp4* and *icp8* in stable cell lines expressing either WT, 6A- or 6D-IFI16-GFP in IFI16-KO HFFs infected with *ICP0-RF* HSV-1 (MOI = 5) measured by RT-qPCR at 6 hpi. Values are means ± SEMs (*n* = 3 or 4). Statistical analysis was performed using ordinary one-way ANOVA. (**B**) Relative RNA transcript levels from *in vitro* T7 transcription reaction containing 5 μM of purified WT or mutant IFI16. 100 ng of FluC control plasmid was used in each reaction as the template. Non-template DNA refers to the genomic DNA isolated from WT HFFs and was added at 1 μg per reaction as indicated. Values are means ± SEMs (*n* = 3). Statistical analysis was performed using ordinary one-way ANOVA. (**C**) Relative mRNA levels of *isg54* and *isg56* in stable cell lines expressing either WT, 6A- or 6D-IFI16-GFP in IFI16-KO HFFs infected with *ICP0-RF* HSV-1 (MOI = 20) measured by RT-qPCR at 6 hpi. Values are means ± SEMs (*n* = 3). Statistical analysis was performed using ordinary one-way ANOVA. (**D**) Progeny virus titers from stable cell lines in (D) infected with *ICP0-RF* HSV-1 virus (MOI = 0.2). Cell-associated and cell-free virus were pooled at 24 hpi or 48 hpi, and the titers of the virus on U-2 OS cells were determined by plaque assay. Values are means ± SEMs (*n* = 3). Statistical analysis was performed using ordinary one-way ANOVA.

Having established a combinatorial role for IFI16 phosphorylations in promoting transition through phase separation stages, we sought to determine their effect on the antiviral functions of IFI16 in either inducing cytokine expression or suppressing viral gene expression. To examine the impact of phosphorylation on viral gene suppression, we infected IFI16 KO, WT, 6A or 6D stable cell lines with *ICP0-RF* HSV-1 and used quantitative reverse transcription PCR (RT-qPCR) to measure the mRNA levels of HSV-1 immediate-early (IE, *icp4*) and early (E, *icp8*) genes at 6 hpi. No major differences in the expression of these IE and E genes were observed in cells expressing either WT or mutant forms of IFI16 at 6 hpi (Figure 5A). To further confirm the retained transcriptional suppression capabilities of the IFI16 phosphomutants, we performed an *in vitro* transcription assay, as in (53). For this assay, we first purified WT-, 6A- and 6D-IFI16-GFP, which we then co-incubated with an *in vitro* transcription reaction using a linearized plasmid containing the firefly luciferase gene (Fluc) under the transcriptional control of T7 promoter. In the presence of WT IFI16, RNA produced from transcription was reduced by ten-fold, indicating that IFI16 is sufficient to suppress transcription *in vitro*. This transcriptional suppression ability was inhibited in the presence of excess non-template double-stranded DNA (Figure 5B), in agreement with previous reports that IFI16 can bind indiscriminately to DNA *in vitro* (15,54). Importantly, both 6A- and 6D-IFI16 were equally capable of suppressing transcription *in vitro*, to an extent similar to the WT, whereas BSA, as a negative control, did not inhibit transcription at the same concentration. These data indicate that IFI16 is sufficient to suppress transcription and that this ability is independent of its phosphorylation state.

We then asked if the capacity of IFI16 to induce cytokine expression was impacted by its phosphorylation state. Using the stable cell lines described above, we performed qPCR to measure the mRNA levels of cytokines (*isg54* and *isg56*) during *ICP0-RF* HSV-1 infection at 6 hpi. We found that cells expressing 6D-IFI16 induced similar levels of cytokines compared to those expressing WT-IFI16. In contrast, cells expressing 6A-IFI16 had dampened cytokine expression (Figure 5C). Consistent with this finding, infected 6A-IFI16 expressing cells had diminished ability to limit viral spread, as seen by the higher virus titer when compared to cells expressing WT or 6D IFI16 at 24 hpi (i.e. after at least one round of virus replication), a phenotype further enhanced at 48 hpi (i.e. after multiple rounds of replication) (Figure 5D).

Overall, these results indicate that IDR phosphorylation does not impact the ability of IFI16 to suppress transcription either *in vitro* or in cells during infection, and that phosphorylation-mediated phase separation of IFI16 specifically promotes cytokine induction.

### CDK2 and GSK3β directly phosphorylate the IFI16 IDR

**Figure 6.**
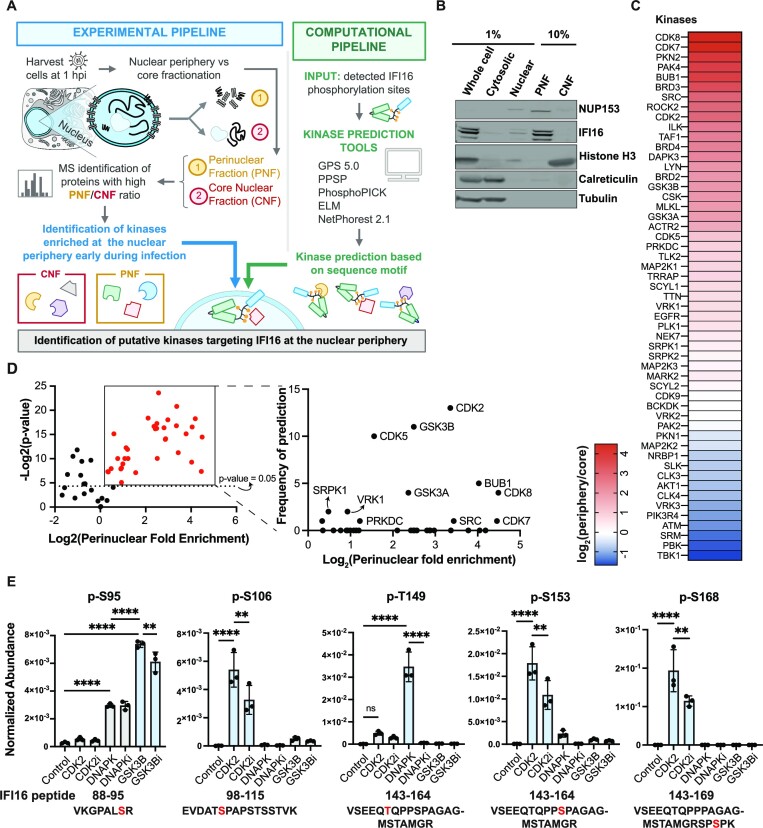
CDK2 and GSK3β phosphorylate multiple sites within the IFI16 IDR. (**A**) Workflow schematic for IFI16 kinase prediction and identification. All experiments were performed in primary HFFs. (**B**) Western blot images of biochemical fractions for indicated proteins. PNF, perinuclear fraction. CNF, core nuclear fraction. (**C**) Heat map showing log_2_ values of ratio of measured group abundances between nuclear periphery and core abundances of all identified kinases. HFF cells were infected with *ICP0-RF* HSV-1 (MOI = 5) and harvested at 1 hpi (*n* = 3). (**D**) Volcano plot showing kinases enriched at perinuclear fractions in (C). Perinuclear fold enrichment for each kinase was calculated as the grouped abundance value of perinuclear fraction over that of core nuclear fraction across three biological replicates. Frequency of prediction by bioinformatics was plotted for kinases with statistically significant (*P*-value < 0.05) perinuclear fold enrichment. (**E**) Normalized abundances for indicated phospho-peptides measured by quantitative mass spectrometry. 4 μg of purified MBP-IFI16-GFP were incubated with Cy5-DNA and 1 μl indicated purified active kinase in the presence or absence of its corresponding inhibitor for 1 h. Samples were then quantified by parallel reaction monitoring (PRM). Values are means ± SEMs (*n* = 3). Samples with corresponding inhibitors added were indicated with ‘i’ appended at the end of the kinase name. Statistical analysis was performed using ordinary one-way ANOVA.

Having determined a role for combinatorial IDR phosphorylation for IFI16 LLPS and antiviral functions, we next asked which kinases target the IFI16 IDR for phosphorylation. We hypothesized that these kinases would localize to the nuclear periphery early in infection. Additionally, such a combinatorial modification status would result either from the action of one kinase phosphorylating multiple sites or of multiple kinases modifying distinct sites. To identify infection- and localization-dependent kinases, we designed a two-pronged approach, integrating biochemical fractionation with computational kinase prediction. Specifically, at 1 hpi with *ICP0-RF* HSV-1, when IFI16 perinuclear puncta form, we separated the nuclear periphery from the nuclear core into distinct fractions (Figure 6A). The effectiveness of this fractionation protocol was confirmed by monitoring the enrichment of nucleoporins and nuclear lamina proteins in the perinuclear fraction (PNF) and of histones and nucleolar proteins in the core nuclear fraction (CNF) using western blot and quantitative mass spectrometry (Figure 6B, S6A and Supplementary Table S2). Next, using mass spectrometry analysis, we identified 34 kinases specifically enriched at the nuclear periphery when compared to the nuclear core fraction at 1 hpi (Figure 6C). These PNF-enriched kinases were further probed using several kinase prediction tools that analyze sequence motifs, focusing on the IDR phosphorylation sites that we tested via mutations (Figure 6A). Overall, 12 kinases passed the two criteria of being enriched in the PNF and bioinformatically predicted as possibly targeting the IFI16 IDR sites (Figure 6D, left). Among these, several cyclin-dependent kinases (particularly CDK2) and the glycogen synthase kinase 3 beta (GSK3β) were the top candidates (Figure 6D, right). Both of these kinases were predicted to phosphorylate multiple IFI16 sites (Supplementary Figure S6B). To experimentally confirm their activity on the IFI16 IDR, we used an *in vitro* kinase assay and designed a targeted mass spectrometry method for the detection and quantification of the IDR phosphorylated peptides. In addition to these top two kinase candidates (CDK2 and GSK3β), we tested the catalytic subunit of DNA-dependent protein kinase (DNA-PKcs, or PRKDC), which we have previously reported to phosphorylate IFI16 at T149 (35) and our current bioinformatics analysis also predicted to act on an additional IDR site (Supplementary Figure S6B). Specifically, we incubated purified IFI16-GFP and DNA with the active form of each purified kinase in the presence or absence of its specific inhibitor. We then subjected the samples to targeted mass spectrometry to relatively quantify the phosphorylated IFI16 IDR peptides. Five of the IDR sites—S95, S106, T149, S153 and S168—were found to be phosphorylated in a kinase activity-dependent manner (Figure 6E, Supplementary Table S3). In agreement with our prior report and our current bioinformatics prediction, DNA-PK phosphorylated the T149 site, acting as a positive control for our *in vitro* assay. Additionally, we discovered that GSK3β phosphorylates the IFI16 S95 site, an activity diminished by treatment with a GSK3β-specific inhibitor, TWS-119. CDK2 was found to modify multiple sites, S106, S153 and S168, an activity reduced upon treatment with a specific inhibitor, CDK2-IN-4.

**Figure 7.**
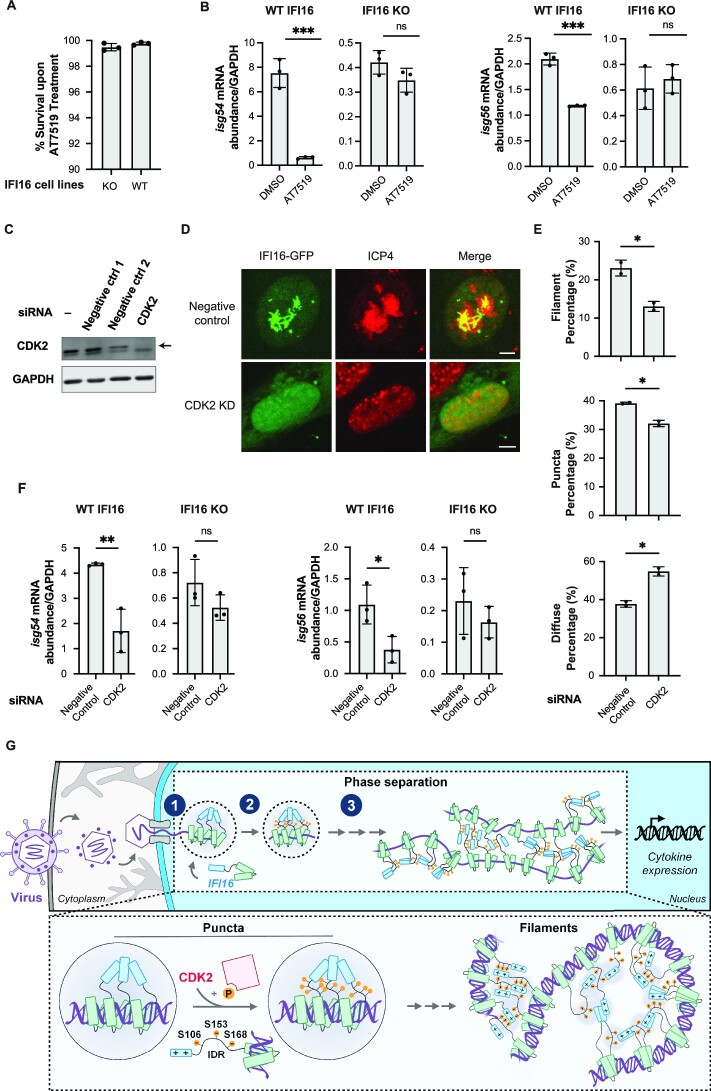
CDK2 knockdown led to reduced IFI16 LLPS and dampened IFI16-dependent cytokine induction. (**A**) Percentage cell survival of HFFs after AT7519 treatment at 5 μM for 6 h. Cell apoptosis was measured using TUNEL assays that measure PARP cleavage, a signature of apoptotic signaling. (**B**) Relative mRNA levels (means ± SEMs, *n* = 3) of *isg54* and *isg56* in IFI16-KO HFF stable cell lines or scrambled-KO HFFs infected with *ICP0-RF* HSV-1 (MOI = 5) and treated with DMSO or 5 μM AT7519, measured by RT-qPCR at 6 hpi. Statistical analysis was performed using unpaired *t*-test. Values are means ± SEMs (*n* = 3). (**C**) Western blot images of indicated samples blotting for CDK2 and GAPDH following siRNA-mediated knockdown in WT HFFs. (**D**) Representative confocal images of IFI16-GFP stably expressing HFFs infected with *ICP0-RF* HSV-1 (MOI = 10) at 8 hpi after negative control or CDK2 knockdown and stained for ICP4. Scale bar, 5 μm. **(E)** Quantification of percentage of cells (N > 100) displaying puncta, filament or diffuse phenotype for conditions described in **(D)**. Values are means ± SEMs (*n* = 2). (**F**) Relative mRNA levels (means ± SEMs, *n* = 3) of *isg54* and *isg56* in IFI16-KO HFF stable cell lines or scrambled-KO HFFs infected with *ICP0-RF* HSV-1 (MOI = 5) after negative control or CDK2 knockdown, measured by RT-qPCR at 6 hpi. Statistical analysis was performed using unpaired *t*-test. Values are means ± SEMs (*n* = 3). (**G**) Model for IFI16 LLPS in immune activation. IFI16 molecules first bind to viral DNA at the nuclear periphery, giving rise to puncta. CDK2 phosphorylates IFI16 IDR at S106, S153 and S168, facilitating LLPS of IFI16. As infection progresses and IFI16 expression increases, IFI16 transitions from a punctate state to solid filamentous state, which is mediated by interactions between positively charged residues within PY and phosphorylated residues within IDR. Finally, LLPS of IFI16 activates downstream signaling pathways to promote cytokine induction.

To determine whether the kinase activities we observed *in vitro* support IFI16-mediated cytokine response, we infected IFI16-KO HFFs or scrambled-KO HFFs with *ICP0-RF* HSV-1 and treated the cells with vehicle control or AT7519, a pan-CDK inhibitor that also inhibits GSK3β (55). Upon verifying that the inhibitor treatment does not promote apoptosis (Figure 7A), we measured the mRNA abundance of interferon-stimulated genes, *isg54* and *isg56*, by RT-qPCR. In the control cells (scrambled-KO HFFs), kinase inhibition led to reduced *isg54* and *isg56* expression levels (Figure 7B). This effect was absent in IFI16-KO HFFs, indicating that the effect of the kinase modulation on cytokine induction is dependent on IFI16. Given our finding that CDK2 phosphorylates multiple IFI16 sites, we next specifically examined the role of CDK2 in regulating IFI16 LLPS and IFI16-dependent cytokine induction in cells. Upon siRNA knockdown of CDK2 (Figure 7C) in IFI16-GFP expressing HFFs, we observed a significant reduction in IFI16 filament formation during infection (Figure 7D, E). Additionally, CDK2 knockdown also led to reduced *isg54* and *isg56* levels in scrambled-KO HFFs, but not in IFI16-KO HFFs during infection (Figure 7F). Altogether, our results demonstrate that CDK2 is present at the nuclear periphery early in HSV-1 infection and induces the phosphorylation of multiple sites within the IFI16 IDR, thereby regulating the induction of cytokine expression.

## DISCUSSION

When facing foreign insults, cells must maintain a delicate balance that allows for an effective immune defense mechanism, while ensuring that the response is not too strong to cause harmful autoimmunity. This is especially critical in the context of nuclear DNA sensing. The nucleus is full of host chromosomes, so the DNA sensors need to distinguish between foreign and host DNA to avoid aberrant spontaneous activation, and at the same time also be tightly regulated to activate specifically in the presence of foreign DNA. For example, aberrant expression of the nuclear DNA sensor IFI16 has been associated with several autoimmune diseases, including Sjögren's syndrome (56), systemic lupus erythematosus (57), systemic sclerosis (58,59), inflammatory bowel disease (60,61), rheumatoid arthritis (58), and psoriasis (62,63). It has also been suggested that IFI16 filamentation with DNA contributes to its autoantigen status in Sjögren's syndrome (64). Thus, investigations into the mechanistic cues that toggle DNA-dependent IFI16 oligomerization during contexts of PRR sensing have important implications not only for understanding antiviral immunity, but also for autoimmune diseases and immunotherapies.

Here, we establish that the innate immune PRR sensor IFI16 undergoes LLPS in the presence of dsDNA *invitro* and *in vivo*. We discovered an intrinsically disordered region on IFI16 that regulates its LLPS capability, and demonstrated that CDK2 acts on multiple sites within the IDR to promote LLPS and cytokine induction (Figure 7G). *In vitro*, we demonstrate that IFI16 LLPS controls the formation of two distinct biophysical modalities, droplets and filaments, in a concentration-dependent manner (Figure 1). In cells, we establish that LLPS governs IFI16-mediated binding to viral DNA genomes and induction of cytokines during HSV-1 infection, thereby restricting virus production. Additionally, we identify an IFI16 IDR, located between the PY and first HIN domain, which we demonstrate to be necessary for initiating LLPS. Taking advantage of the ability offered by *Ciona intestinalis* to visualize the dynamics of phase separating proteins (34), we obtain *in vivo* evidence for the IDR requirement for IFI16 puncta and filament formation. By manipulating IDR sequences through deletions and reconstitutions, we abolish and restore IFI16 aggregation during different stages of embryonic development within *Ciona*. We find that at the core of this IDR function in LLPS is a combinatorial effect of multiple phosphorylation sites. We establish that phosphorylation does not impact the initial recruitment of IFI16 to viral genomes, but rather its progression through stages of LLPS from puncta to filaments as infection progresses. The phosphorylation-mediated phase separation in turn promotes IFI16-mediated cytokine expression and limits virus spread (Figures 3 and 4).

Our IFI16 phosphorylation mutant studies showed that diminished phase separation inhibited the ability of IFI16 to induce cytokine expressions, but not to suppress viral gene expression early in infection (Figure 5). Thus, the phosphorylation-mediated phase separation uncouples these two antiviral functions of IFI16, which were previously frequently considered to work in tandem. It is tempting to propose a model in which LLPS of IFI16 provides the needed biophysical properties to recruit and activate downstream signaling factors, such as those involved in the STING-TBK1-IRF3 axis, to trigger the induction of antiviral cytokines. On the other hand, the suppression of virus gene expression by IFI16 may occur through mechanisms independent of LLPS, such as the direct recruitment of transcriptional suppressors to the site of viral transcription through protein-protein interactions (14,20,24,65,66). Our *in vitro* transcription assay suggests that, even in the absence of other transcriptional suppressors, IFI16 is sufficient for transcriptional suppression *in vitro* (Figure 5B). This suggests a model where IFI16 could suppress transcription through occupancy of the DNA, possibly by inhibiting the recruitment of the RNA polymerase. An alternative explanation for this would be that IFI16 could “fold" the DNA through the oligomerization of multiple IFI16 molecules occupying different regions of the DNA across long distances, thus making transcription elongation inefficient. While future studies are still required to differentiate these mechanisms, our study establishes the sufficiency of IFI16 to suppress transcription *in vitro*. Altogether, the IFI16 functional decoupling mutants we generated can serve as valuable tools for dissecting downstream antiviral pathways of IFI16, which could be conserved across other innate immune factors that perform one or both functions similar to IFI16.

The LLPS capability of IFI16 could also be linked to its reported role in inflammasome formation, which was previously shown to involve ASC and caspase 1 in the context of Kaposi's sarcoma-associated herpesvirus (KSHV) infection in macrophages and endothelial cells (12), as well as of HSV-1 infection in macrophages and fibroblasts (67,68). Similar to NLRP6 inflammasome activation, which has been shown to be driven by double-stranded RNA induced LLPS (69), IFI16 could also activate inflammasome formation through its LLPS capability upon DNA binding. Future studies would be required to characterize a possible link between IFI16 LLPS and its role in inflammasome activation.

The requirement of phosphorylation to promote LLPS indicates that specific kinase(s) for IFI16 must also be present at or in close proximity to the site of viral genome deposition. Through biochemical fractionation, proteomics, and bioinformatics studies, we found that CDK2 and GSK3β are present at the nuclear periphery early in HSV-1 infection and phosphorylate IFI16 at multiple sites within the IDR (Figure 6). Importantly, CDK2 knockdown reduced IFI16 filament formation at later stages of infection (8 hpi; Figure 7C) and dampened the induction of cytokines in an IFI16-dependent manner (Figure 7D). As the solid-state filament formation of IFI16 likely represents a biochemically irreversible process (44) (Figure 1G) and thus a committed step of IFI16 activation, these results suggest CDK2 as a potential licensing factor for the activation of IFI16. This model also highlights the multi-layered regulation that is required for DNA sensors in the nucleus, in a manner analogous to the co-activation model for T cell receptors (70). While the heterochromatin state of the host DNA could still be the predominant way by which IFI16 autoimmune activation is regulated (16), the discovery of kinases targeting IFI16 and the regulation of the kinases themselves provide another mechanism by which IFI16 can be inhibited. Specifically, the heterochromatinization state of the host DNA could dictate that self-DNA-bound IFI16 remains at a low local concentration below the threshold of LLPS. In contrast, the phosphorylation within the IFI16 IDR may act as a safeguard mechanism by ensuring that this threshold remains high (Figure 1G). This may be more relevant during cell cycle progression in S phase, when nascent DNA is synthesized but is yet to be packaged with nucleosomes. Kinase misregulation could thus potentially lead to aberrant IFI16 LLPS and immune activation. Indeed, increased activities of CDK enzymes have been associated with heightened inflammation in activated macrophages (71). Additionally, elevated CDK2 abundance was found in sera of patients with the autoimmune disease Pemphigus Vulgaris, with CDK2 inhibition limiting the development of pathologies in a mouse model system of this disease (72). On the other hand, GSK3β has been implicated in Sjögren's syndrome, an autoimmune disease associated with IFI16 overexpression and autoantibodies against IFI16 filaments (73).

With the functional relevance of IFI16 filaments established, one important consideration is how they are mechanistically assembled. Here, we demonstrate that the positively charged residues within the PY act synergistically with the phosphorylated residues within the IDR to promote IFI16 filamentation, since we showed that either domain alone was insufficient for filament formation (Figure 4F) and loss of charge in either domain results in a failure to form IFI16 filaments in cells (Figure 4G and H). Given these findings, we propose a model in which IFI16 filamentation is driven by multivalent interactions between PY and IDR, thus allowing for a mesh-like binding of IFI16 that gives rise to filaments (Figure 7G).

Taken together, our findings point to LLPS as a key biophysical prerequisite to initiate IFI16 antiviral functions. The requirement for a threshold in both concentration and phosphorylation levels enables a switch-like behavior for the activation of IFI16 to carry out its antiviral functions. These findings also have important mechanistic implications for mammalian PY-containing proteins with potential IDRs immediately C-terminal of the PY, most of which have reported roles in modulating immunity (e.g. NLRPs, ASC, and PYHIN proteins AIM2, IFIX) (5,74–78). This study also adds to the accumulating evidences for a broader role for LLPS in innate immunity. For example, in the cytoplasm, the DNA sensor cGAS forms spherical liquid condensates that are critical for its DNA sensing capacity (41,47). Here, we uncover the relevance of LLPS in the context of nuclear DNA sensing, thus further expanding the link between LLPS and the regulation of innate immunity. Of interest is also the different relationship between phosphorylation and LLPS in these contexts of cytoplasmic or nuclear sensing. While cGAS phosphorylation suppresses LLPS to prevent activation during mitosis (47), IFI16 phosphorylation within the IDR acts to promote LLPS and cytokine induction. This distinction highlights the divergent roles of phosphorylations in regulating LLPS. By extension, it is also possible for IFI16 to be inhibited through toggling between different phosphorylation states during mitosis to prevent autoactivation. Future studies can help determine whether the phosphorylation sites characterized in this study or other sites may also regulate IFI16 activity during mitosis.

Altogether, our study links phosphorylation-driven LLPS with nuclear DNA sensing. Our findings provide a mechanistic explanation for how IFI16 switch-like phase transitions, including dynamic oligomeric puncta and stable filamentation, are achieved with high temporal and spatial resolution for immune signaling initiation and maintenance. The IFI16 LLPS, as dictated by its phosphorylation status, can serve as a biophysical rheostat that toggles phases of innate immune activity. These results also promise to provide valuable insights into the regulation of autoimmunity.

## Supplementary Material

gkad449_Supplemental_Files

## Data Availability

The mass spectrometry proteomics data have been deposited to the ProteomeXchange Consortium via the PRIDE (32) partner repository with the dataset identifier PXD034348 and 10.6019/PXD034348. All PRM data has been deposited to Panorama Public (33): https://panoramaweb.org/IFI16_LLPS_PRM.url. The RAW data and a compiled peptide library have been uploaded to ProteomeXchange (identifier PXD034348).
