# Supplementary material for: IFI16 phase separation via multi-phosphorylation drives innate immune signaling
Source: Nucleic Acids Res. 2023 Jun 7;51(13):6819–40. doi: 10.1093/nar/gkad449 (PMC10359621; doi:10.1093/nar/gkad449)
Supplement: gkad449_Supplemental_Files [file gkad449_supplemental_files.zip › Supplemental Text and Figures.pdf]

MOVIES

Movie 1. *IFI16 displays dynamic puncta formation at the nuclear periphery at early HSV-1 infection.*

IFI16-eGFP expressed in the nuclei of HFFs after infection with WT HSV-1 (MOI = 10) at 1 hpi. Images were taken at 2 min per frame for HSV-1. Scale bar, 5 μm.

Movie 2. *3D reconstruction of MBP-IFI16-GFP with Cy5-DNA.*10 μM of purified MBP-IFI16-GFP incubated with equimolar Cy5-DNA for 1 hour. Scale bar, 5 μm.

Movie 3. *IFI16-GFP in Ciona embryos throughout gastrula stages.*

Maximum intensity projection of ectodermal cells of Ciona embryos expressing IFI16-GFP (in green) and H2b-mCherry (in magenta) imaging starts at the early gastrula stage at 31 seconds per frame.

Movie 4. *FUS-IFI16-GFP in Ciona embryos throughout gastrula stages.*

Maximum intensity projection of ectodermal cells of Ciona embryos expressing FUS-IFI16-GFP (in green) and H2b-mCherry (in magenta) imaging starts at the early gastrula stage at 31 seconds per frame.

TABLES

Table S1. *PRM quantification of IFI16 phospho-peptides in IFI16-GFP IP in primary HFFs infected by ICP0-RF HSV-1, at 1 and 6 hpi.*

Table S2. *Normalized abundances in PNF and CNF for proteins detected in DDA in primary HFFs infected by ICP0-RF HSV-1, at 1 hpi.*

Table S3. *PRM quantification of IFI16 phospho-peptides in in vitro kinase assay.*

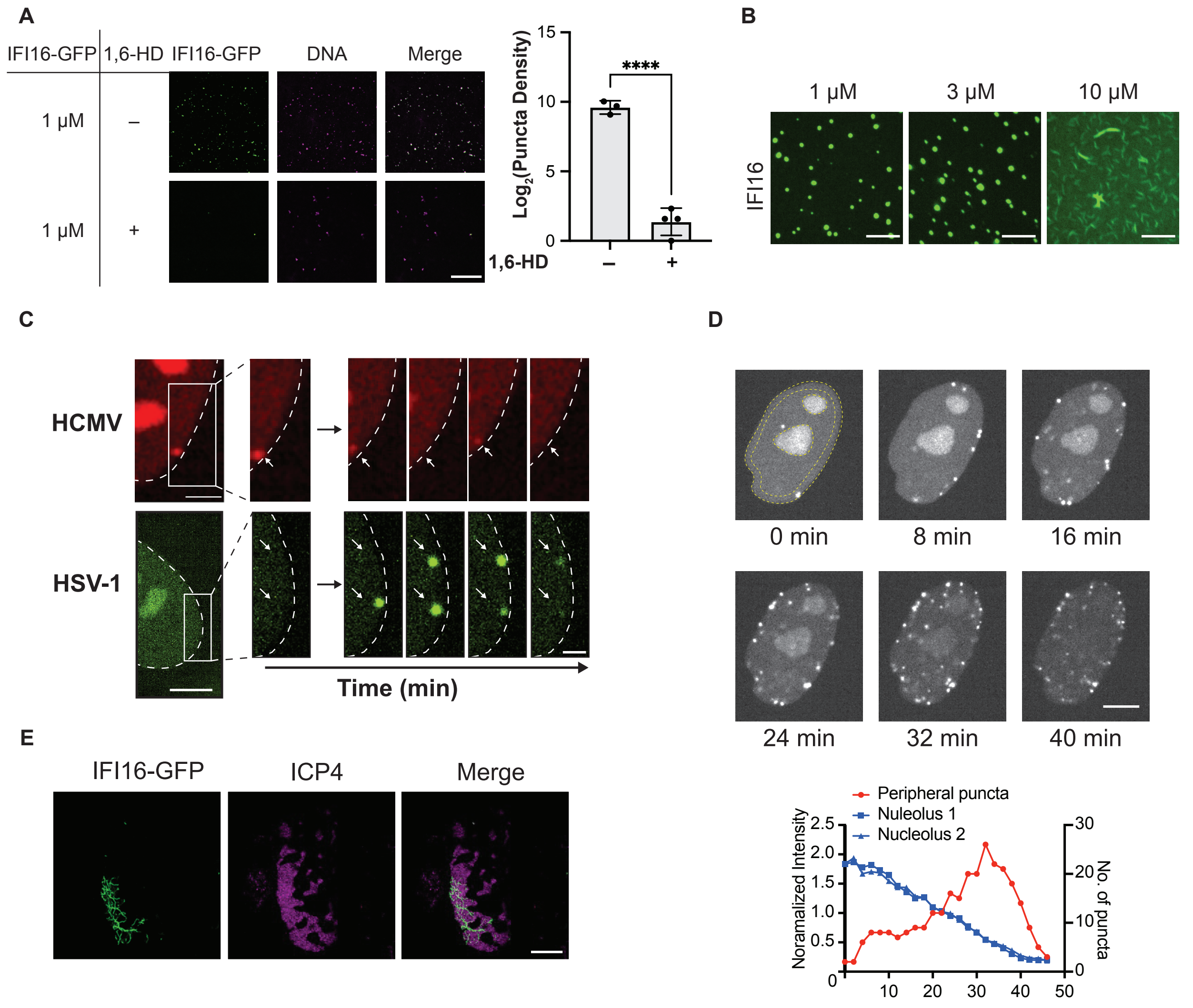

Figure S1. *IFI16 moves from nucleolus to nuclear periphery to form dynamic puncta during HSV-1 infection.*

- **A.** Representative images and quantification of IFI16-GFP LLPS at 1 μM in the presence or absence of 10% 1,6-HD. Scale bar, 5 μm. Puncta density was calculated as the number of IFI16 puncta visualized per field. Statistical analysis was performed using unpaired t-test. Quantification was shown for one representative replicate out of three performed. Values are means ± SEMs (n = 3).
- **B.** Representative images of IFI16-GFP LLPS at the indicated concentrations in the presence of equimolar Cy5-DNA. Scale bar, 5 μm.
- **C.** IFI16-FusionRed (top) or IFI16-eGFP (bottom) stably expressed in the nuclei of HFFs after infection with WT HCMV (MOI = 3, top) or WT HSV-1 (MOI = 10, bottom) at 1 hpi. Images were taken at 4 min per frame for HCMV and 2 min per frame for HSV-1. Scale bar, 5 μm.
- **D.** Nuclear IFI16-eGFP stably expressed in HFFs time-lapse images during early infection with WT HSV-1 (MOI = 10, 1 hpi). Scale bar, 5 μm. Three regions of interest (ROIs) representing the nuclear periphery and two nucleoli are outlined in yellow. Mean gray values of ROIs are measured using Fiji and normalized by the average intensity value across all frames. Normalized intensities are plotted for the three ROI across all frames.
- **E.** Confocal images of HFFs stably expressing IFI16-GFP infected with *ICP0-RF* HSV-1 (MOI = 10, 8 hpi) and stained for ICP4. Scale bar, 5 μm.

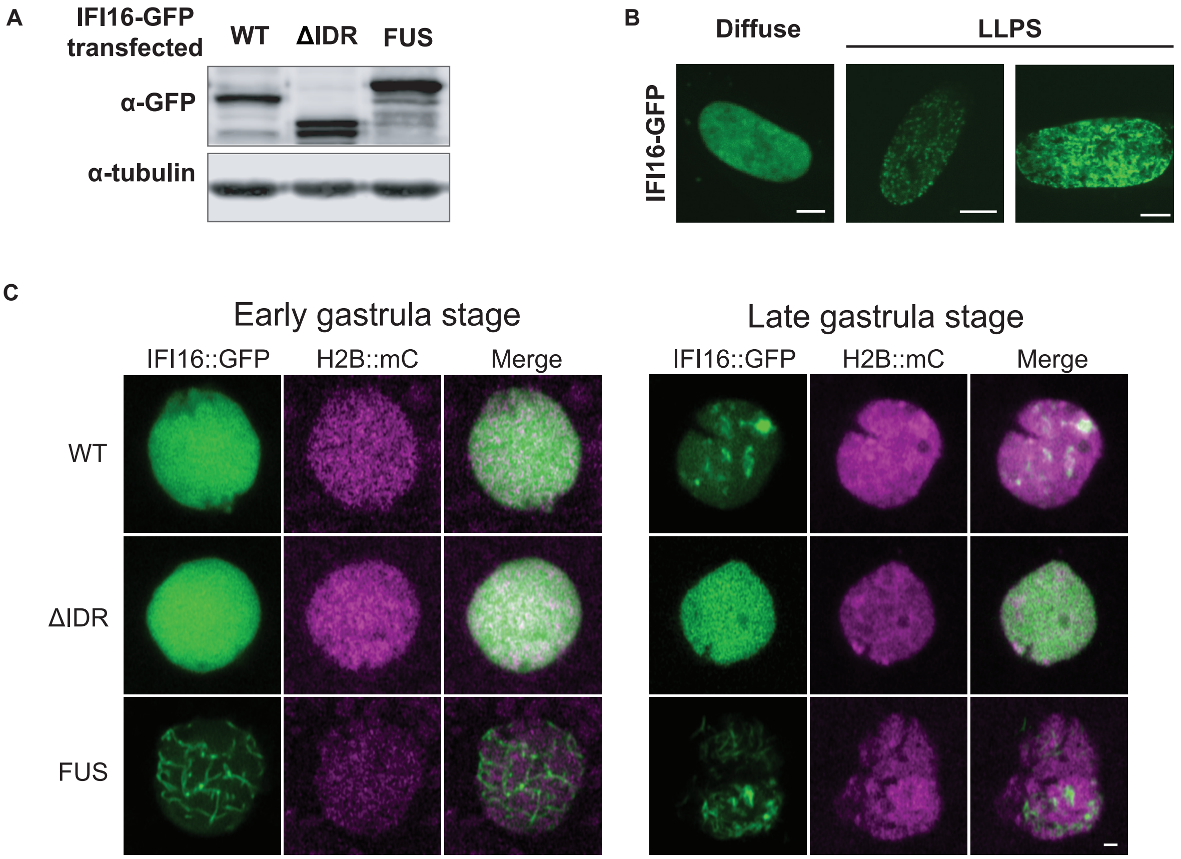

Figure S2. *The IDR is required for aggregate formation of IFI16 in Ciona embryos.*

- **A.** Western blot images of HEK293T-STING cells transfected with the indicated IFI16-GFP constructs and blotted for GFP and tubulin.
- **B.** Example images for diffuse and LLPS phenotypes of IFI16-GFP transiently expressed in WT HFFs. Scale bar, 5 μm.
- **C.** Single confocal sections of *Ciona* nuclei at the early and late gastrula stages. IFI16::GFP has a uniform distribution at the early gastrula stage and forms aggregates at the late gastrula stage. IFI16::IDR showed uniform distributions at all observed stages, and IFI16::FUS::GFP showed a filamentous distribution from the early gastrula stage. Scale bar, 1 μm.

A

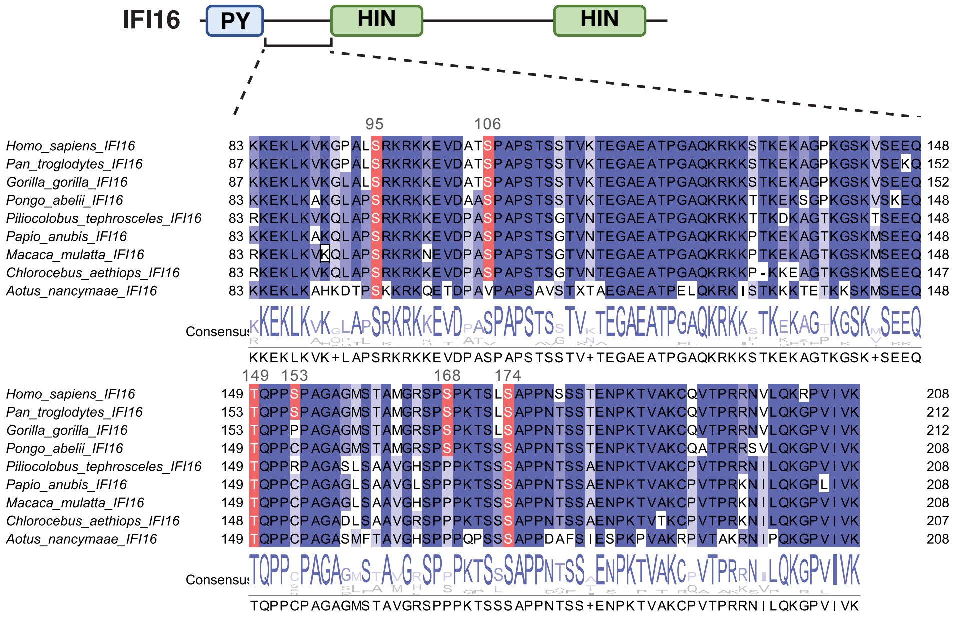

B

Species	Common Name	Isoform	S95	S106	T149	S153	S168	S174
Homo sapiens	human	2	S	S	T	S	S	S
Pan troglodytes	chimpanzee	X1	S	S	T	S	S	S
Gorilla gorilla	gorilla	X3	S	S	T	P	S	S
Pongo abelii	Sumatran orangutan	8	S	S	T	C	S	S
Macaca mulatta	rhesus monkey		S	S	T	C	P	S
Papio anubis	olive baboon	X1	S	S	T	C	P	S
Chlorocebus aethiops	African green monkey		S	S	T	C	P	S
Piliocolobus tephrosceles	Ugandan red colobus	X1	S	S	T	R	P	S
Aotus nancymaae	Ma's night monkey		S	V	T	C	P	S

Figure S3. *Conservation of phosphorylation sites within the IFI16 IDR in primate species.*

A. Schematic of IFI16 and alignment of IDR (aa 83-208) region with other primate species. Phosphorylation sites for which point mutations were generated are colored in pink.

B. Highlighted phosphorylation sites and their conservation in other primate species. Sites mutated in other species are colored in red.

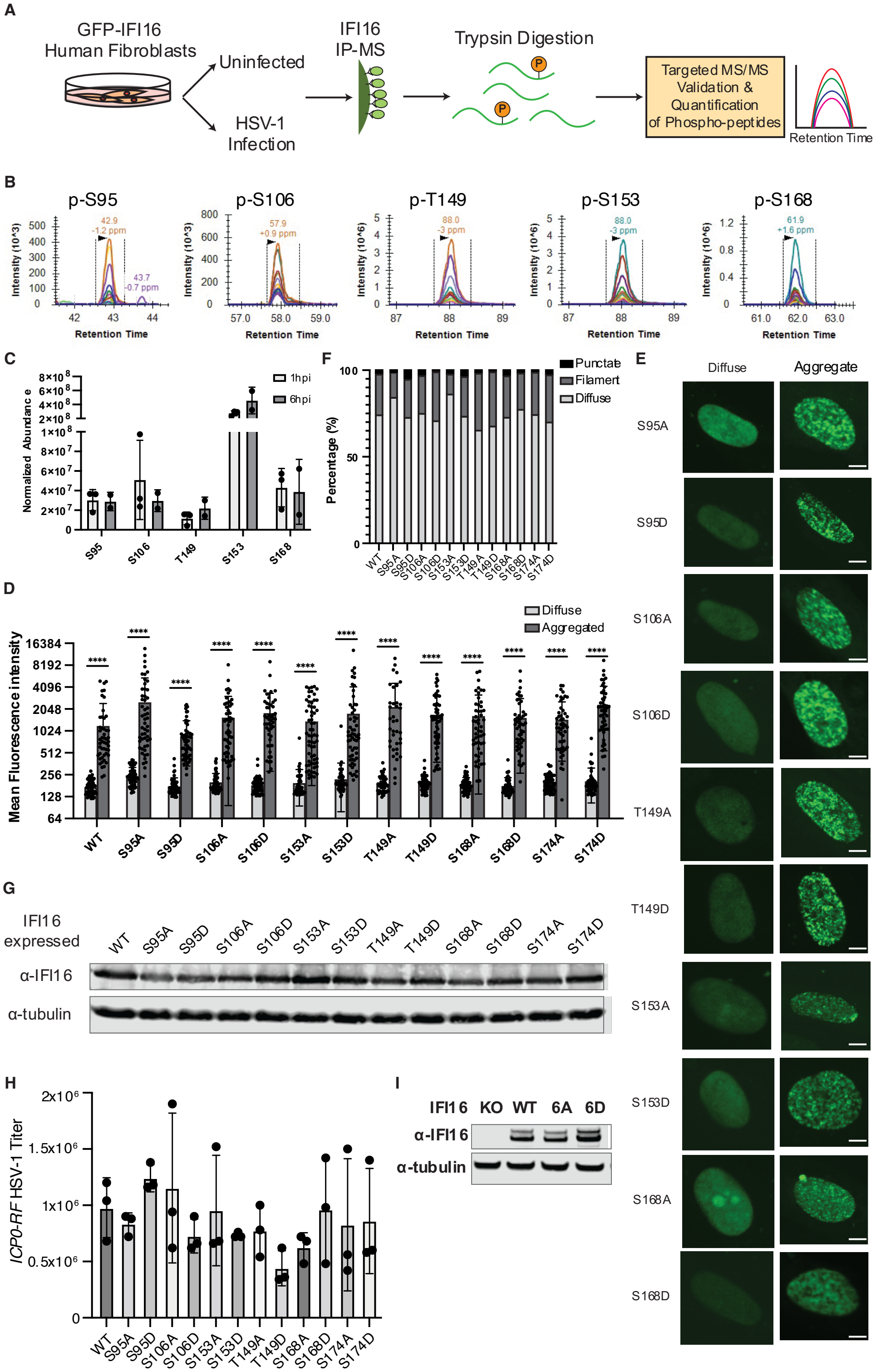

Figure S4. *Single phosphorylation sites within the IDR are not sufficient to promote IFI16 LLPS and antiviral immunity.*

- **A.** Schematic showing the workflow for immunoaffinity purification-targeted mass spectrometry (PRM). Experiments were performed in WT HFFs.

B. Representative extracted fragment ion chromatograms (n ≥ 4 co-eluting fragments) are shown for IFI16 modified tryptic peptides that were validated in 3 biological replicates in HFFs infected with *ICP0-RF* HSV-1 (MOI = 5, 1 hpi).

C. Normalized abundances (mean ± SEM, n = 3) of indicated phosphopeptides at 1 hpi and 6 hpi. All tryptic peptides were normalized to the abundance value of an unmodifiable IFI16 peptide.

D. Mean fluorescent intensities of single phosphomutants of IFI16-eGFP transiently expressed in WT HFFs, grouped by diffuse or aggregated phenotypes (N=100 for each group).

E. Representative images of single phosphomutants of IFI16-eGFP transiently expressed in WT HFFs. Each IFI16 single phosphomutant retains the ability to form aggregates in cells.

F. Quantification of percentage of cells (N>100) displaying filament, punctate or diffuse phenotypes of WT or mutant IFI16-GFP transiently expressed in WT HFFs.

G. Western blots showing the expression levels of IFI16-eGFP transiently expressed in HEK293T-STING cells.

H. Progeny virus titers from HEK293T-STING cells transfected with indicated plasmids and infected at 24 hours post-transfection with ICP0-RF HSV-1 virus (MOI = 0.2). Cell-associated and cell-free virus were pooled at 24 hpi, and the titers of the virus on U2OS cells were determined by plaque assay. Values are means ± SEMs (n = 3).

I. Western blot images of stable cell lines expressing WT, 6A- or 6D-IFI16-GFP in IFI16-KO HFFs

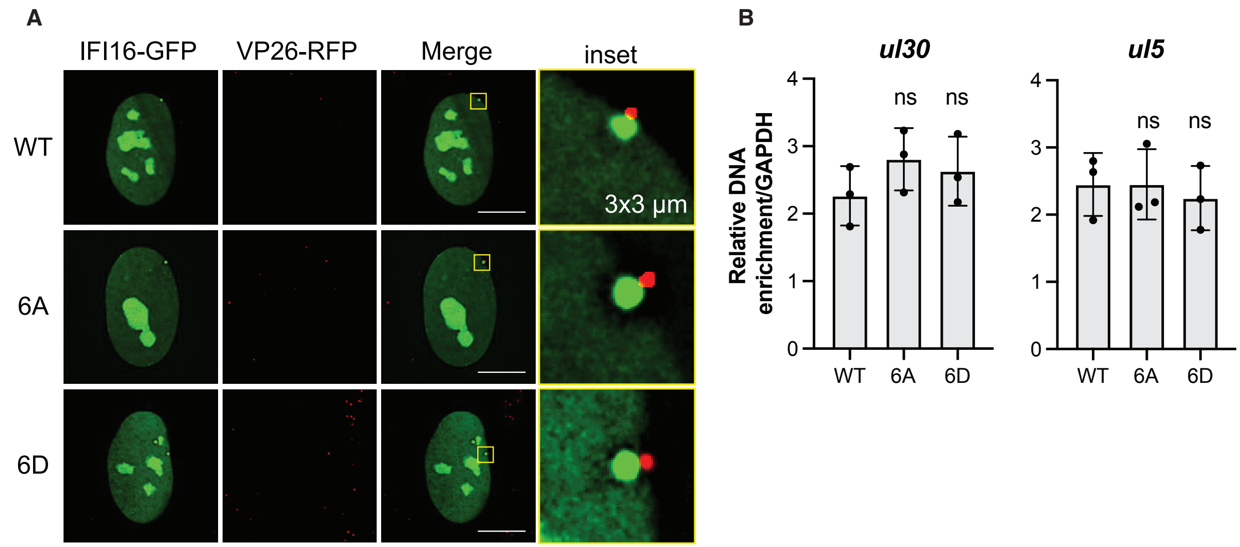

Figure S5. *Phosphorylation of IFI16 does not impact its recruitment to viral genomes.*

- **A.** Representative images of cell lines stably expressing either WT, 6A- or 6D-IFI16-GFP in IFI16-KO HFFs during infection with HSV-1::rfp-vp26 (MOI 10, 2 hpi). Scale bar, 10 μm.
- **B.** Relative DNA levels of ul30 and ul5 enriched after ChIP-qPCR performed in stable cell lines expressing either WT, 6A- or 6D-IFI16-GFP in IFI16-KO HFFs infected with ICP0-RF HSV-1 (MOI = 1, 2 hpi). Values are means ± SEMs (n = 3). Statistical analysis was performed using ordinary one-way ANOVA.

A

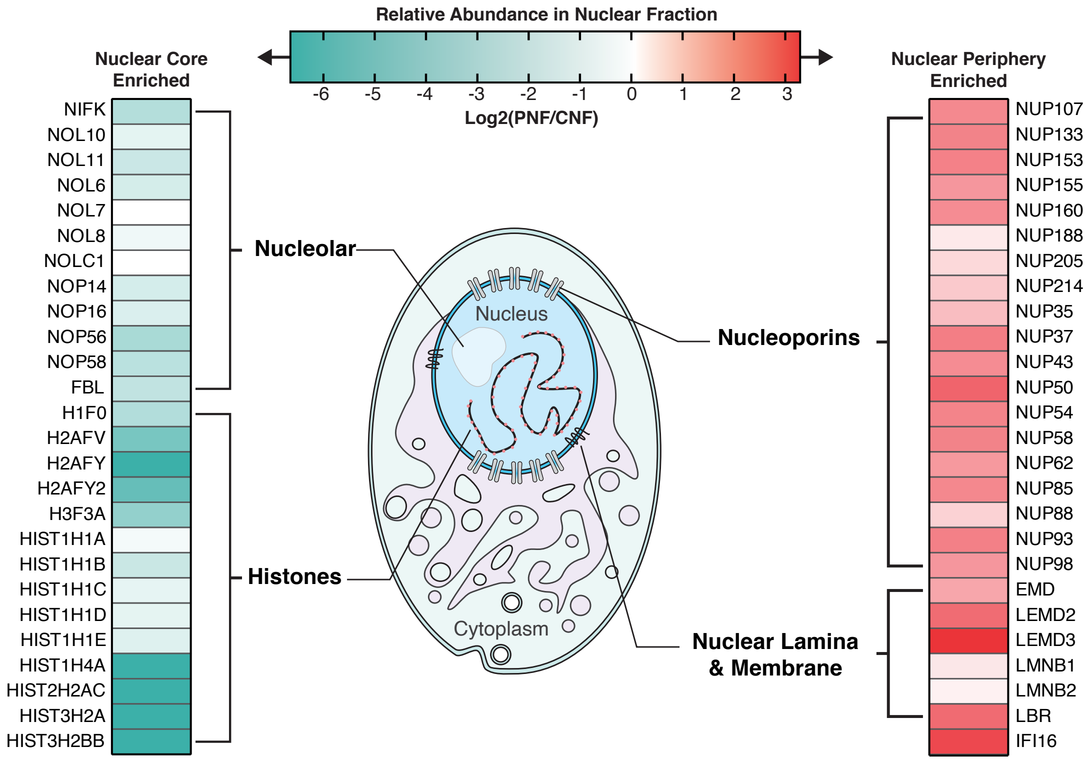

B

Kinase	S95	S106	T149	S153	S168	S174
CDK2	1	4	0	3	4	1
GSK3B	1	3	1	3	3	1
CDK5	1	2	0	3	3	1
BUB1	1	1	0	1	1	1
CDK8	0	0	0	2	2	0
GSK3A	0	1	1	1	1	1
VRK1	1	0	0	1	0	0
SRPK1	0	0	0	0	1	1
CDK7	0	0	0	0	1	0
SRC	0	0	0	0	1	0
PRKDC	0	0	1	1	0	0
MAP2K3	0	0	0	0	0	1
ILK	0	0	1	0	0	0

Figure S6. *Nuclear periphery and core fractionation validation and kinase prediction.*

A. Representative heat map showing relative abundance values for proteins known to be localized in the nuclear periphery/core (n = 3). Experiments were performed in WT HFFs.

B. Table showing all perinuclear-enriched kinases and their respective frequencies (calculated as the sum of instances the kinase is predicted) of predictions by bioinformatics on the indicated phosphorylation sites of IFI16.
